# Codon usage bias regulates gene expression and protein conformation in yeast expression system *P. pastoris*

**DOI:** 10.1186/s12934-021-01580-9

**Published:** 2021-04-26

**Authors:** Yichun Xu, Kunshan Liu, Yu Han, Yanzi Xing, Yuanxing Zhang, Qiuying Yang, Mian Zhou

**Affiliations:** 1 State Key Laboratory of Bioreactor Engineering, East China University of Science and Technology, Shanghai, 200237 China; 2 State Key Laboratory of Biocatalysis and Enzyme Engineering, School of Life Sciences, Hubei University, Hubei, 430062 China

**Keywords:** Codon bias, *P. pastoris*, Protein expression, Conformation

## Abstract

**Background:**

Protein synthesis is one of the extremely important anabolic pathways in the yeast expression system *Pichia pastoris*. Codon optimization is a commonly adopted strategy for improved protein expression, although unexpected failures did appear sometimes waiting for further exploration. Recently codon bias has been studied to regulate protein folding and activity in many other organisms.

**Results:**

Here the codon bias profile of *P. pastoris* genome was examined first and a direct correlation between codon translation efficiency and usage frequency was identified. By manipulating the codon choices of both endogenous and heterologous signal peptides, secretion abilities of N-terminal signal peptides were shown to be tolerant towards codon changes. Then two gene candidates with different levels of structural disorder were studied, and full-length codon optimization was found to affect their expression profiles differentially. Finally, more evidences were provided to support possible protein conformation change brought by codon optimization in structurally disordered proteins.

**Conclusion:**

Our results suggest that codon bias regulates gene expression by modulating several factors including transcription and translation efficiency, protein folding and activity. Because of sequences difference, the extent of affection may be gene specific. For some genes, special codon optimization strategy should be adopted to ensure appropriate expression and conformation.

**Supplementary Information:**

The online version contains supplementary material available at 10.1186/s12934-021-01580-9.

## Introduction

In the nuclear genome, there are 64 codons coding for 20 amino acids plus three stop codons. Therefore, there is obviously a redundancy. Amino acids except Met and Trp are coded with 2–6 synonymous codons, and the codon selection in coding sequences (CDS) is not random. This is considered as codon usage bias, which appears in almost all genomes [1]. For a long time, codon usage bias has been believed to regulate the rate of protein synthesis [2]. More frequent codons are used in highly expressed genes, which help to accelerate translation rate because of the high abundance of the decoding tRNAs [3]. Oppositely, rare codons slow down protein translation and always accumulate in lowly expressed genes [3, 4].

Besides translation rate, recently codon usage bias has been reported to participate in multiple levels of regulation including translation accuracy [5], co-translational folding [6–8] and transcription [9]. Our previously published data suggests that wild-type codon choice maintained the correct structure and function of the *Neurospora* circadian clock component FRQ, which was then eliminated by codon optimization [8]. The similar phenotype was observed on *Drosophila* circadian clock protein PERIOD [7] and cyanobacteria clock protein KaiBC [8]. In many genomes, a negative correlation between codon usage bias and regional secondary structure was observed [10]. Codon usage bias has also been proven to affect gene transcription recently. For luciferase gene expressed in *Neurospora*, altered nucleotide sequence through codon optimization resulted in elevated transcription efficiency and decreased H3K9 tri-methylation levels [11]. Besides, codon choice is an important factor to regulate mRNA stability [12] as well as premature transcription termination (PTT) [9]. However, more solid evidences in multiple organisms are still needed.

Codons in the very 5′ region of CDS are thought to have greater importance. For the previously mentioned clock genes (*Neurospora frq* and *Drosophila dper*), N-terminal codon optimization was enough to cause impaired protein function on circadian behavioral rhythms [8, 9]. In *Escherichia coli*, N-terminal rare codons were found to increase the expression of green fluorescent protein by at most 14-fold [13]. The existence of a “ramp” with 30–50 slowly translated codons at the 5′ CDS was confirmed in many prokaryotic and eukaryotic organisms [4]. As for the mechanism of how ramp works, there is an argument between avoidance of ribosomal traffic jam [4] and reduced mRNA secondary structure [13]. It is true that in most eukaryotic species the nucleotide at the third position of rare codons is A or U, which contributes to weaker secondary structures. However, in some other species such as methylotrophic yeast *Pichia pastoris*, frequent codons are rich in A/U (Additional file 1: Table S1). Therefore, the effect of N-terminal rare codons on protein expression becomes a more complicated issue.

Moreover, 5′ CDS is also a region encoding signal sequence such as secretive peptide and organelle localization signal [14, 15]. A strong preference of non-optimal codons has been found in signal sequences [16–18]. In *E. coli*, when non-optimal codons in signal peptides were replaced by optimal codons, the expression and export of mature maltose-binding protein (MBP) [19] and β-lactamase [20] were influenced. However, a systematic study on the role of codon usage bias of signal sequences in regulating protein expression and secretion in eukaryotes is still missing.

The yeast *P. pastoris* has become a widely used expression system for proteins and secondary metabolites in the past 20 years. *P. pastoris* has numerous advantages including stronger ability to bear unfolded protein response [21, 22], highly efficient methanol-induced promoter P_*AOX1*_ [23], easy genetic manipulation [24] and high density fermentation [25]. In most cases proteins are secreted due to its strong secretion ability. The development of synthetic biology recently prompted researches focusing on chassis cells construction supported by promoter engineering [26–28] and metabolic remodeling [29, 30]. However, not enough attention has been paid towards codon optimization, especially its mechanism in regulating protein synthesis in *P. pastoris* expression system. Traditional codon optimization replaces rare codons with frequent codons to a certain extent, maintaining the overall GC content within a normal range. By this way, many protein products were successfully expressed [31–34] while others failed in experimental trials. Due to the distinct AU rich codon preference in *P. pastoris*, it is necessary to study the role of codon usage bias in regulating protein secretion and folding in this system. Mechanistic study will also help to generate updated optimizing strategies.

Here in this study, the codon preference in *P. pastoris* genome was examined first. Then 4 endogenous signal sequences as well as the α-mating factor [35] were focused on to study the role of their codon usage bias on whole protein expression and secretion. After that, the roles of extreme codon optimization were studied on 2 disordered proteins. In addition to the traditional degradation assay, Circular Dichroism (CD) was performed to compare structural differences between purified proteins with and without codon optimization.

## Materials and methods

### Strains and culture conditions

*E. coli* Top 10 cells were used for plasmid construction and propagation. Top 10 cells were cultured in LLB medium, containing 0.5% yeast extarct, 1% tryptone and 0.5% NaCl at 37 °C, and 100 µg/ml of ampicillin or 50 µg/ml of Zeocin was added to the medium when required.

*Pichia pastoris* strain GS115 was used as the wild-type and the host to make transgenic strains. All cells were shaking cultured at 220 rpm, 30 °C. For seed preparation, yeast cells were inoculated into YPD medium (2% tryptone, 1% yeast extract, 2% glucose) until OD_600_ reached 6–8. If with *GAP* promoter, cells were then inoculated into YPD again with initial OD_600_ 1.0. If with *AOX1* promoter, cells were washed three times by sorbitol or sterile water and inoculated into BMMY medium (2% tryptone, 1% yeast extract, 1.34% YNB, 1% methanol, 0.1 M potassium phosphate buffer) with initial OD_600_ 1.0 for induction. 0.5% methanol was supplemented every 24 h.

### Plasmid construction and strain generation

All codon optimized or de-optimized sequences were synthesized by Genewiz. The α-amylase sequence with C-terminal 6 × His tag was amplified from existing constructs in our group [21]. The original sequences of *PAS_chr2-2_0376* and *PAS_chr2-2_0432* were amplified from the *P. pastoris* genome.

For SPs and α-mating factor, original/optimized/de-optimized sequences were ligated with α-amylase and pPIC9k backbone by a ClonExpress MultiS one step cloning kit (Vazyme) to generate pPIC9k-*sp9ori-AmyA*, pPIC9k-*sp9deopt-AmyA*, pPIC9k-*sp9opt-AmyA*, pPIC9k-*sp20ori-AmyA*, pPIC9k-*sp20deopt-AmyA*, pPIC9k-*sp20opt-AmyA*, pPIC9k-*sp13ori-AmyA*, pPIC9k-*sp13deopt-AmyA*, pPIC9k-*sp13opt-AmyA*, pPIC9k-*sp15ori-AmyA*, pPIC9k-*sp15deopt-AmyA*, pPIC9k-*sp15opt-AmyA* pPIC9k-*aFori-AmyA*, pPIC9k-*aFdeopt-AmyA*, pPIC9k-*aFoptN-AmyA*, pPIC9k-*aFoptC-AmyA* and pPIC9k-*aFopt-AmyA,* pPIC9k-*sp23ori-AmyA*, pPIC9k-*sp23deopt-AmyA*, pPIC9k-*sp23opt-AmyA*, pPIC9k-*sp34ori-AmyA*, pPIC9k-*sp34deopt-AmyA*, pPIC9k-*sp34opt-AmyA*. Each construct was linearized inside *his4* by SalI and electroporated into the GS115 wild-type strain. Positive transformants were selected on histidine deficient plates and strains with a single copy of transgene were verified by PCR.

For *PAS_chr2-2_0376* and *PAS_chr2-2_0432*, both original and codon optimized sequences were ligated into pGAPZA after the *GAP* promoter to generate pGAPZA-*0376ori*, pGAPZA-*0376opt*, pGAPZA-*0432ori*, pGAPZA-*0432opt* and pGAPZA-*0432optC*. The C-terminal c-Myc and 6 × His tag were kept for easier western blot detection. Each construct was then linearized inside *GAP* promoter by *Avr*II and electroporated into GS115 wild-type strain. Positive transformants were selected on Zeocin containing plates and strains with a single copy of transgene were verified by PCR.

For 0376 protein purification, original and optimized sequences of *0376* were also ligated into pPICZα and pPICZB after the *AOX1* promoter to generate pPICZα-*0376ori*, pPICZα-*0376opt*, pPICZB-*0376ori* and pPICZB-*0376opt*. The C-terminal c-Myc and 6 × His tag were kept for easier purification. Each construct was then linearized inside *AOX1* promoter by *Pme*I and electroporated into GS115 wild-type strain. Positive transformants were selected on Zeocin high concentration plates (0.7 mg/mL) and strains with the highest target protein expression were selected by Western blot.

### Data resources and codon bias analysis

The genomic information of *P. pastoris* GS115 was retrieved from NCBI database (https://www.ncbi.nlm.nih.gov/genome). Codon usage frequency was downloaded from the Codon Usage Database (http://www.kazusa.or.jp/codon/). The indicated data of *S. cerevisiae* was retrieved from Frydman’s Lab (http://www.stanford.edu/group/frydman/codons) [36]. tAI values were calculated according to Reis et.al [37], and ω values and CAI were calculated as described by Sharp et al. [38]. Protein structural disorder score was calculated by IUPred (https://iupred2a.elte.hu/). A sliding window size of 5 codons for α-mating factor signal peptide and 10 codons for *PAS_chr2-2_0376*, *PAS_chr2-2_0432* was used when plotting CAI curves. Correlation between codon usage and disorder tendency were calculated as described previously [10].

For extreme codon optimization, synonymous codons with highest ω value were used to replace original codons. For de-optimization, synonymous codons with lowest value were used to replace original codons (Additional file 1: Tables S1, S3, S4).

### Amylase activity assay

50 mL cell culture was harvested at indicated time points and OD_600_ was measured. Samples were then centrifuged to separate cells and supernatant. For secretive expression, supernatant was collected. For intracellular expression, cellular proteins were extracted by mechanical disruption [29]. The enzyme activity of amylase was measured by the dinitrosalicylic acid (DNS) method [26]. Specific enzyme activity (U/OD) was calculated by dividing measured activity by OD_600_.

### Western blot

50 mL cell culture was harvested at indicated time points and OD_600_ was measured. Samples were then centrifuged to separate cells and supernatant. For intracellular expression, cellular proteins were extracted by mechanical disruption [29] and 50 µg total protein was loaded. For secretive expression, appropriate amount of supernatant normalized by OD_600_ was loaded to ensure comparability. Western blot was performed as described [6]. Primary antibody was 6 × His tag antibody (Beyotime) or β-Actin antibody (Abcam), and secondary antibody was HRP conjugated (Beyotime). Band intensities were quantified by Image J.

### Quantitative real-time PCR

50 mL cell culture was harvested at indicated time points and cells were pelleted by centrifugation. Total RNA was extracted by Yeast RNA extraction kit (RN10, Aidlab Biotechnologies). For each sample, 2 µg total RNA was used for cDNA synthesis (FastKing RT Kit, TIANGEN) and subsequent quantitative PCR (SYBR Green, TIANGEN). Primers for amplification were designed by the help of Beacon designer 7.9 [26].

### Trypsin sensitivity assay

50 mL cell culture was harvested at indicated time points and cells were pelleted by centrifugation. Intracellular proteins were extracted by mechanical disruption [29] and diluted to a concentration of 7 mg/mL. 150 µL protein extracts were treated with trypsin (final concentration 1 µg/mL) at 25 °C. A 20 uL sample was taken from the reaction at indicated time points, boiled with protein loading buffer immediately and resolved on a SDS-PAGE gel. Western blot was performed to examine target protein levels.

### Protein purification

0376ori and 0376opt proteins were purified by affinity chromatography. 200 mL cell culture was harvested 2-3 days after methanol induction and cells were pelleted by centrifugation. Intracellular proteins were extracted by mechanical disruption [29] by Cobalt Resin Extraction buffer (50 mM Na_3_PO_4_, 300 mM NaCl, 5% glycerol, 0.1% triton, pH = 7.4). Protein extract was then purified with HisPur™ Cobalt Resin (Thermo) after adjusting pH to 7.4 with 1 M NaOH. The resin was washed with Cobalt Resin Wash Buffer (50 mM Na_3_PO_4_, 300 mM NaCl, 10 mM imidazole, pH 7.4) and eluted with Cobalt Resin Elution Buffer (50 mM Na_3_PO_4_, 300 mM NaCl, 150 mM imidazole, pH 7.4). Proteins in each fraction were checked by SDS-PAGE and Coomassie blue staining.

### Circular Dichroism

Eluted proteins were dialyzed with 10 mM Na_3_PO_4_ (pH = 7.4) for circular dichroism spectroscopy and then concentrated to 70 µg/mL by ultrafiltration. The CD spectra were recorded using a Chirascan Circular Dichroism Spectrometer (Applied Photophysics Ltd) with a 1 mm path cell. For melting curves, temperature was raised from 25 to 95 °C with a rate of 5 °C every 5 min. For denature curves, different concentrations of urea were added in to the sample. 2 repetitive scans were obtained for each sample.

## Results

### Examine the codon usage bias in *P. pastoris* genome

**Fig. 1:**
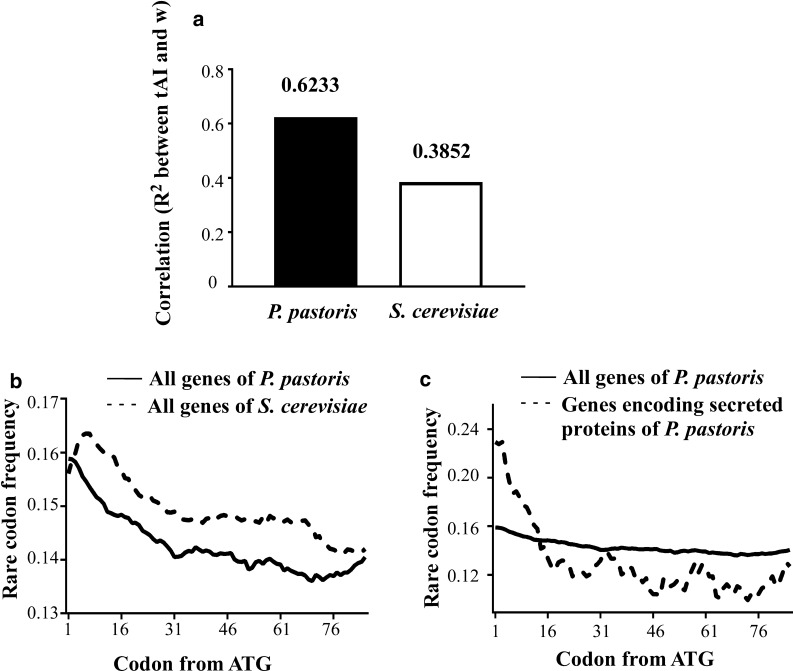
The codon bias profile in *P. pastoris* genome. **a** Correlation between ω and tAI values in *P. pastoris* and *S. cerevisiae*. **b** Rare codon frequency at the 5′ end of all protein coding genes in *P. pastoris* (continuous line) and *S. cerevisiae* (dashed line). **c** Rare codon frequency at the 5′ end of genes coding for secreted proteins (dashed line) and all protein coding genes (continuous line) in *P. pastoris*

Several parameters are used to represent codon usage bias in a genome. Codon adaptation index (CAI) [38] is calculated from relative codon usage frequency (ω), which suggests the chance of a codon being chosen by highly expressed genes. tRNA adaptation index (tAI) [36] is based on tRNA copy number, and therefore tRNA abundance. tAI mainly describes the chance of a tRNA arriving at the A site among all tRNAs carrying the same amino acid. Here ω and tAI values of *P. pastoris* codons were calculated and shown in Additional file 1: Tables S1 and S2 (see “Materials and methods”), and the correlation between them was calculated (Fig. 1a). The correlation (R^2^ = 0.6233) was much higher than budding yeast *S. cerevisiae* which was only 0.3852, as well as many other microbial species [37]. This suggests that *P. pastoris* has a better tune up between codon usage frequency and decoding rate. The rare codon “ramp” was then checked to see whether it also existed in *P. pastoris* CDSs or not. For *S. cerevisiae* and *P. pastoris*, 15 codons with the lowest tAI were defined as rare codons [16]. As shown by Fig. 1b, a narrow ramp (approximately 10–20 codons) marked by higher rare codon frequency was observed at 5′ ends of *P. pastoris* CDSs. Compared with *S. cerevisiae*, the position of ramp showed a small shift towards the initial codon. Although short, the existence of ramp may be necessary and worth further exploration.

### The secretion ability of N-terminal signal peptides is tolerant towards synonymous codon changes

**Fig. 2:**
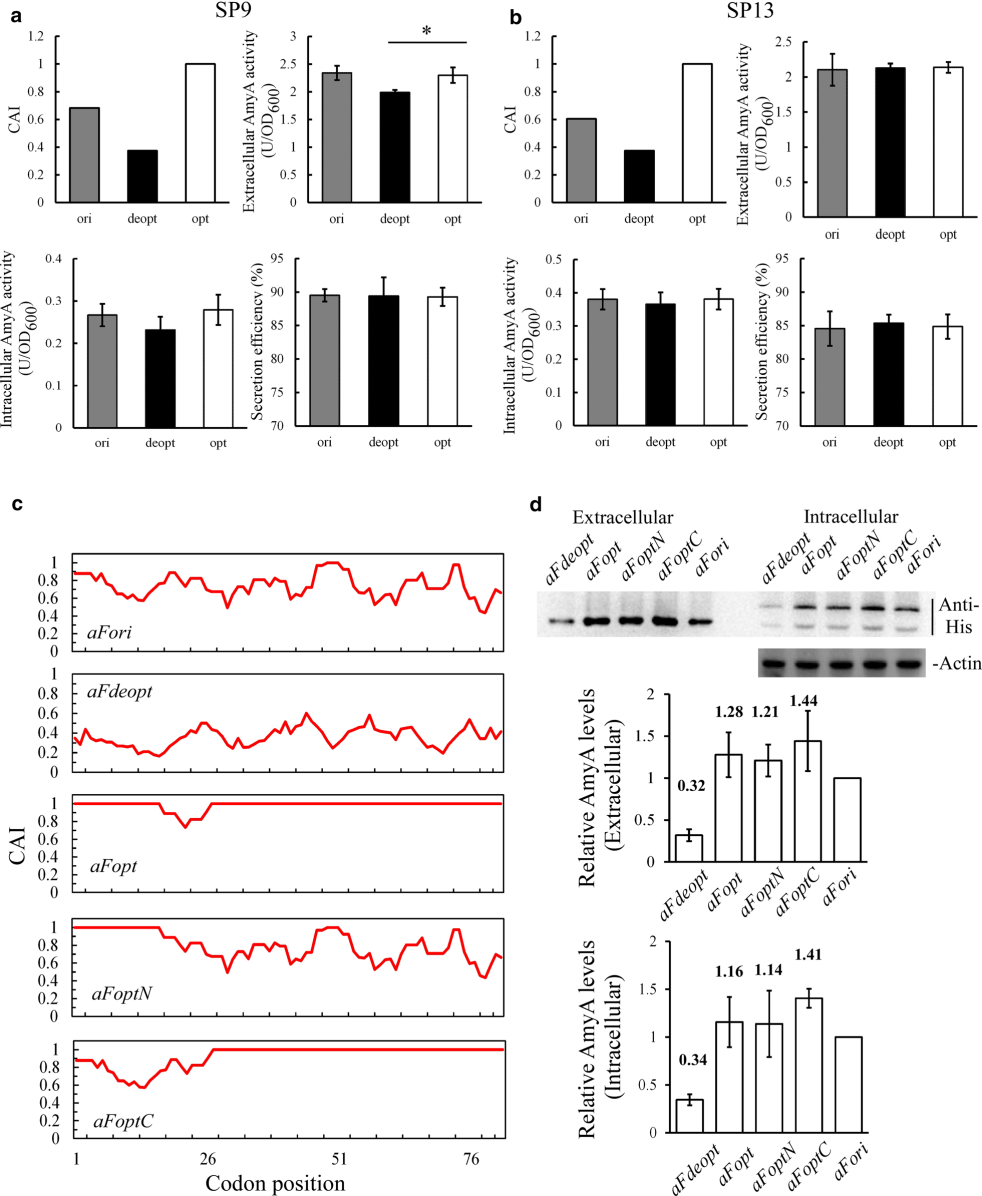
Synonymous codon replacement does not affect the secretion ability of signal peptides. CAI, extracellular & intracellular specific activity of amylase reporter, as well as secretion ability of SP9 (**a**) and SP13 (**b**) when codons were de-optimized or optimized. **c** CAI plots of codon de-optimized and optimized α-mating factor sequences. **d** Top, western blow showing protein levels of extracellular & intracellular protein levels of amylase reporter in codon de-optimized and optimized strains; middle and bottom, densitometric quantification of western blot results. 6 × His tag antibody and β-Actin antibody were used as primary antibodies to detect AmyA and β-actin (loading control), respectively. The level of aFori-AmyA was set as 1. For enzyme activity and western blot quantification, error bars were calculated as standard deviation of three independent experiments. *, p < 0.05

Many signal peptides (SPs) are located at N-terminus of a protein, among which the secretory signal is the most prevalent. According to published study on *P. pastoris* secretome [39], CAI curves of the 39 endogenous secretory SPs were plotted (Additional file 1: Figure S1). Most of these SPs seemed not to adopt optimal codons, with apparent roughs on their CAI curves. When rare codon frequency was plotted in genes coding for secreted proteins, they showed a stronger “ramp” than genome average (Fig. 1c). Six SPs were then picked (SP9 from *PAS_chr1-1_0267*, SP13 from *PAS_chr1-3_0229*, SP15 from *PAS_chr1-3_0226,* SP20 from *PAS_chr4_0643*, SP23 from *PAS_chr1-1_0160* and SP34 from *PAS_chr2-1_0787*) to study whether the codon choice of signal peptides affects the downstream gene expression and secretion. There signal peptide sequences were optimized or de-optimized, and fused with the alpha-amylase gene (*AmyA*) reporter to examine the consequence. Codon optimization increased CAI to 1, while de-optimization lowered CAI significantly below 0.5 (Fig. 2a, b, Additional file 1: Figure S2, top-left panels). After methanol induction for 24 h, both extracellular and intracellular specific activity of AmyA were examined (Fig. 2a, b, Additional file 1: Figure S2, top-right panels and bottom-left panels). The secretion efficiency was also calculated [40] by the extracellular amount divided by total (Fig. 2a, b, Additional file 1: Figure S2, bottom-right panels). Compared with codon optimization, codon de-optimization was slightly associated with decreased extracellular specific activity, with statistical significance in a few cases (SP9, SP15, SP34). However, codon choice of the six SPs did not affect secretion efficiency significantly.

Considering that the twenty-codon long signal peptide occupied only a tiny portion of the whole protein, it may be too short to bring a phenotype. The 86 amino acid long α-mating factor signal peptide was then studied in addition to the endogenous signal peptides. Derived from budding yeast *S. cerevisiae*, this peptide is now prevalently used in *P. pastoris* system for recombinant protein expression and secretion. However, little work has been done to reveal whether its codon design is possible to regulate the ability of secretion. The original sequence of α-mating factor signal peptide shows many troughs when CAI was calculated according to *P. pastoris* genome (Fig. 2c, top panel). Based on the original sequence (*aFori*), completely de-optimized α-mating factor signal peptide sequence (*aFdeopt*) and completely optimized one (*aFopt*) were designed. Since the α-mating factor prepro-peptide is composed by two regions: a 19 amino acid long pre-peptide followed by a 67 amino acid long pro-peptide [35], two more codon-optimized sequences *aFoptN* (first 19 codons optimized) and *aFoptC* (last 67 codons optimized) were then made. CAI curves after codon optimized are plotted in Fig. 2c. Again, *AmyA* [26] was fused with these sequences to be a reporter. Different aF-AmyA genes were transformed into GS115 strains separately to make single copy GS115-aF-AmyA strains. Instead of enzyme activity, here we used western blot to quantify intracellular and extracellular alpha-amylase levels more directly. As shown by Fig. 2d, codon de-optimization of α-mating factor signal peptide largely decreased both intracellular and extracellular protein levels to around 30%, while codon optimization elevated the levels by 20–40%. However, the extracellular and intracellular protein ratios compared with original sequence were comparable among different codon manipulated strains. Together, the above results from endogenous signal peptides and α-mating factor prepro-peptide suggest that the secretion function of most signal peptides is tolerant towards synonymous codon changes.

### Full-length codon optimization on structurally disordered proteins affects their expression patterns

**Fig. 3:**
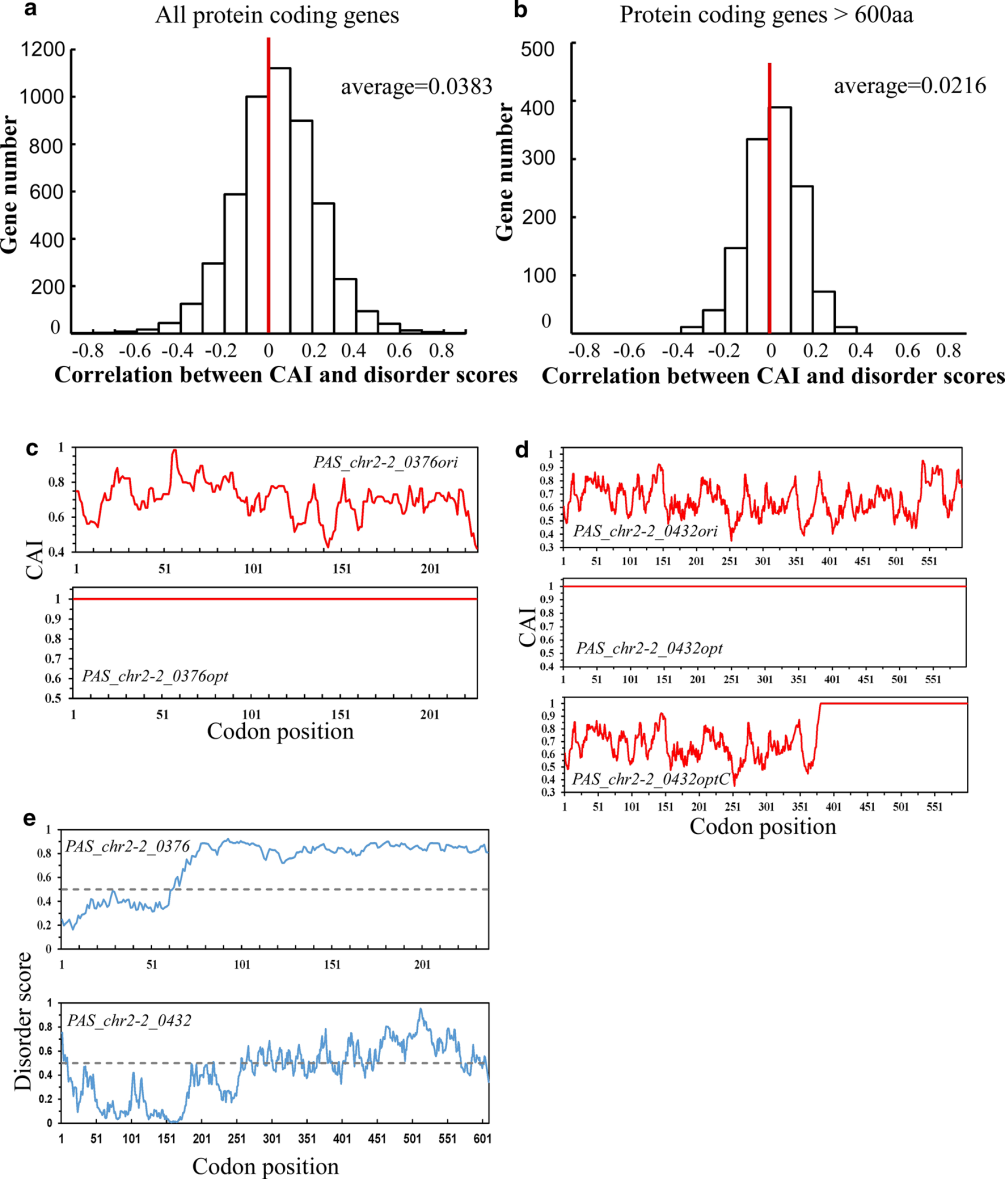
Codon usage bias are negatively correlated with protein structural disorder in a significant number of *P. pastoris* genes. **a** The distribution of CAI and disorder score correlation of all protein coding genes in *P. pastoris*. **b** The distribution of CAI and disorder score correlation of protein coding genes longer than 600aa in *P. pastoris*. **c**, **d**, CAI score plot of *PAS_chr2-2_0376*, *PAS_chr2-2_0432* and their codon optimized sequences, respectively. **e** Disorder score plot of *PAS_chr2-2_0376* and *PAS_chr2-2_0432*

Previous bio-statistical analyses performed in the genomes of *N. crassa, S. cerevisiae and C. elegans* revealed a negative correlation between codon bias and structural disorder in most protein coding genes [10]. Therefore a similar genome-wide analysis in *P. pastoris* was performed here. Surprisingly, most protein coding genes did not show a strong bias towards a positive or negative correlation (Fig. 3a). A similar result was got when only large ORFs (> 600 aa) were included in analyses (Fig. 3b).

**Fig. 4:**
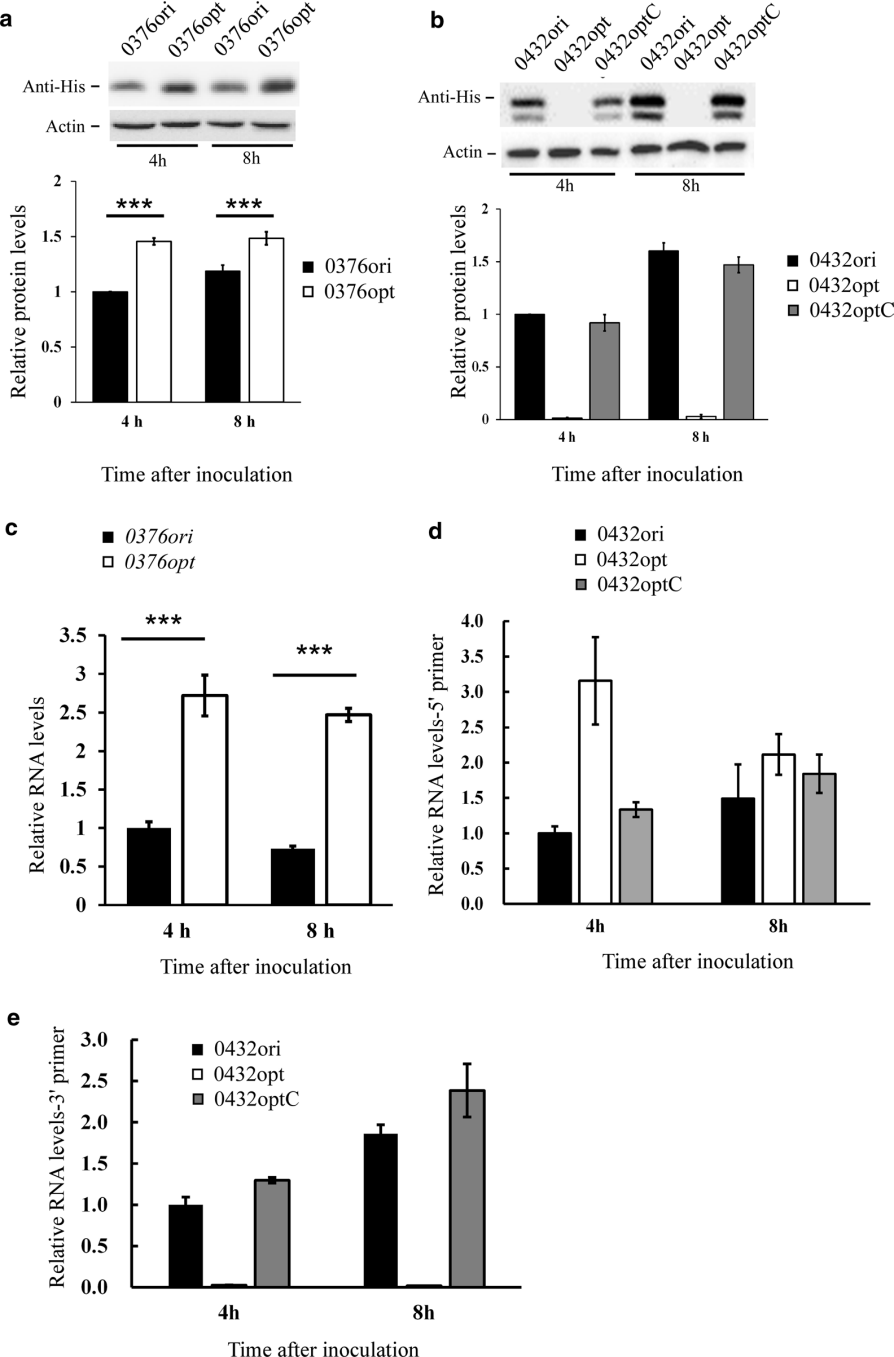
The expression profiles of two gene candidates after codon optimization. **a**, **b** Top, western blot showing protein levels of *PAS_chr2-2_0376* (**a**), *PAS_chr2-2_0432* (**b**) before and after codon optimization; bottom, densitometric quantification of western blot results. 6 × His tag antibody and β-Actin antibody were used as primary antibodies to detect target proteins and β-actin (loading control), respectively. **c** qPCR showing RNA levels of *PAS_chr2-2_0376* before and after codon optimization. **d**, **e**, qPCR showing RNA levels of *PAS_chr2-2_0432* before and after codon optimization with 5′ primer sets (**d**) and 3′ primer sets (**e**). Error bars were calculated as standard deviation of three independent experiments. *, *p* < 0.05; **, *p* < 0.01; ***, *p* < 0.001

Based on our previous studies [11], structurally disordered proteins may be more fragile towards codon optimization. Two gene candidates with different levels of structural disorder were then picked to examine their performance under full-length codon optimization. The two genes are *PAS_chr2-2_0376* (disorder score = 0.656, mentioned as *0376* later) and *PAS_chr2-2_0432* (disorder score = 0.332, mentioned as *0432* later). CAI curves before and after codon optimization are shown in Fig. 3c, d and structural disorder curves are plotted in Fig. 3e. All CAI and disorder scores are also summarized in Table 1. Both original (ori) and codon optimized (opt) sequences were introduced into the *GS115* wild-type cells separately to make single copy transgenic strains. A 6 × His tag was added after the ORF to distinguish them from the endogenous gene. Western blot was then performed to compare indicated protein levels before and after codon optimization. The protein level of 0376 was elevated by 30–50% after codon optimization (Fig. 4a). However, we failed to detect any proteins in codon optimized *0432* strains (Fig. 4b).

**Table 1:** CAI, correlation and disorder scores of *PAS_chr2-2_0376* and *PAS_chr2-2_0432*

	*0376*	*0376opt*	*0432*	0432opt	*0432C* (codon381-609)
CAI	0.685	1	0.643	1	0.646
Disorder	0.656	0.656	0.332	0.332	0.600
Correlation between CAI and disorder	− 0.122	–	− 0.244	–	− 0.191

To further address this question, *0432* mRNA levels were examined by qPCR. Primer sets were designed from the 5′ and 3′ UTR regions to maintain the same amplification efficiency between ori and opt sequences, and exclude the interference of endogenous sequence. Interestingly, 5′ *0432* mRNA level was comparable or higher in the *0432opt* strain while the 3′ mRNA was largely abolished (Fig. 4d, e). A C-terminal codon optimized sequence was then designed (Fig. 3e, bottom panel, mentioned as *0432optC* later). Again transgenic yeast strains were made and protein as well as mRNA levels were examined. As a result, both 0432 protein (Fig. 4b) and 3′ mRNA levels were rescued (Fig. 4e). The dual bands of 0432 protein were possibly caused by alternative initiation during translation or partial degradation. Taken together, the expression failure of *0432opt* may be a result of premature transcription termination or extreme 3′ RNA instability caused by A/U rich synonymous codon replacement. This problem was circumvented when only the C-terminus was optimized.

### Codon optimization alters protein trypsin sensitivity and stability under freeze–thaw cycles

**Fig. 5:**
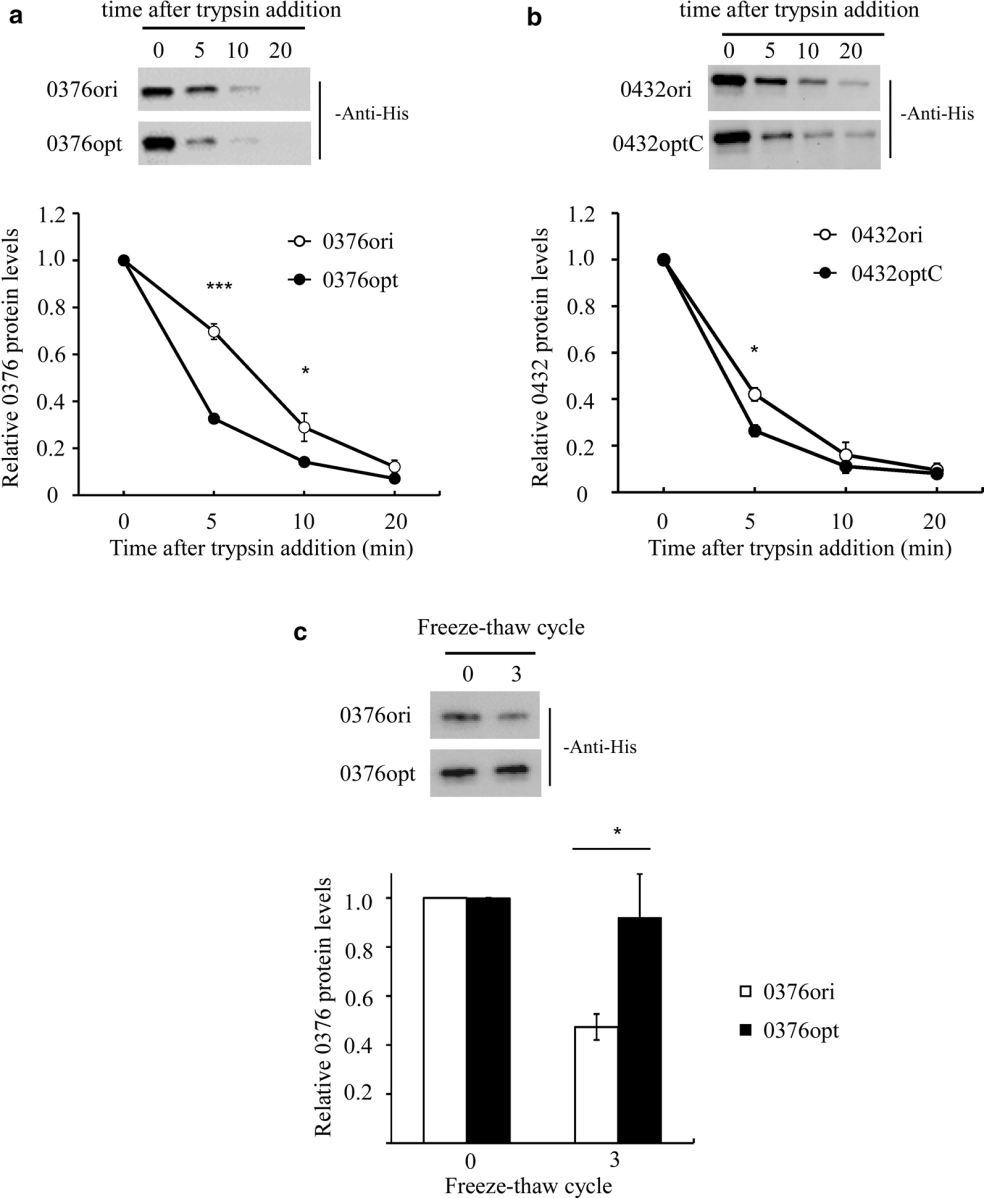
Codon optimization altered protein stability upon trypsin digestion and freeze–thaw cycles. **a**, **b** Top, western blot showing 0376 (**a**) and 0432 (**b**) protein sensitivity towards trypsin digestion before and after codon optimization; bottom, densitometric analyses of the western blot results. The protein levels at time point 0 were set as 1. **c** Top, western blot slowing 0376 protein degradation after freeze and thaw cycles before and after codon optimization; bottom, densitometric analyses of the western blot results. The protein levels at freeze–thaw cycle 0 were set as 1. 6 × His tag antibody was used as primary antibody to detect target protein. Error bars were calculated as standard deviation of three independent experiments. *, *p* < 0.05; **, *p* < 0.01; ***, *p* < 0.001

Although the 0376 protein expression was successfully improved by codon optimization, 30–50% was not a satisfied ratio to us since codon bias was known to increase protein levels by at least three to four folds in many examples [6, 11]. Besides, RNA analysis suggested that *0376* mRNA level was elevated to two to three folds by codon optimization (Fig. 4d), and this difference seemed to shrink a lot at the protein level. Our previous study on *N. crassa* FRQ protein suggested that codon optimization of structurally disordered protein regions affected folding and function. To test whether this was also true in *P. pastoris*, we then checked the stability of 0376 towards trypsin digestion to see whether codon optimization resulted in any conformation change. As shown by Fig. 5a, proteins translated from codon optimized *0376* started with a higher level but went through a faster decay rate after trypsin treatment. Trypsin digestion assay was also performed on *0432ori* and *0432optC* strains. Again, 0432optC protein was more sensitive towards trypsin digestion compared with 0432ori (Fig. 5b), although the difference was smaller than that in 0376 group. This was probably due to shorter optimized regions and weaker structural disorder in 0432. Together, these results indicate that trypsin sensitivity of 0376 and 0432 proteins were increased upon codon optimization, suggesting altered conformations.

Besides trypsin sensitivity, the 0376 protein stability was also tested under freeze and thaw cycles. As shown by Fig. 5c, 0376opt was quite stable after three freeze–thaw cycles while 0376ori was degraded by half. Again, this suggests that 0376 protein structure was altered by codon optimization by a certain extent.

### Codon optimization alters protein CD spectroscopy profiles upon temperature and urea treatment

**Fig. 6:**
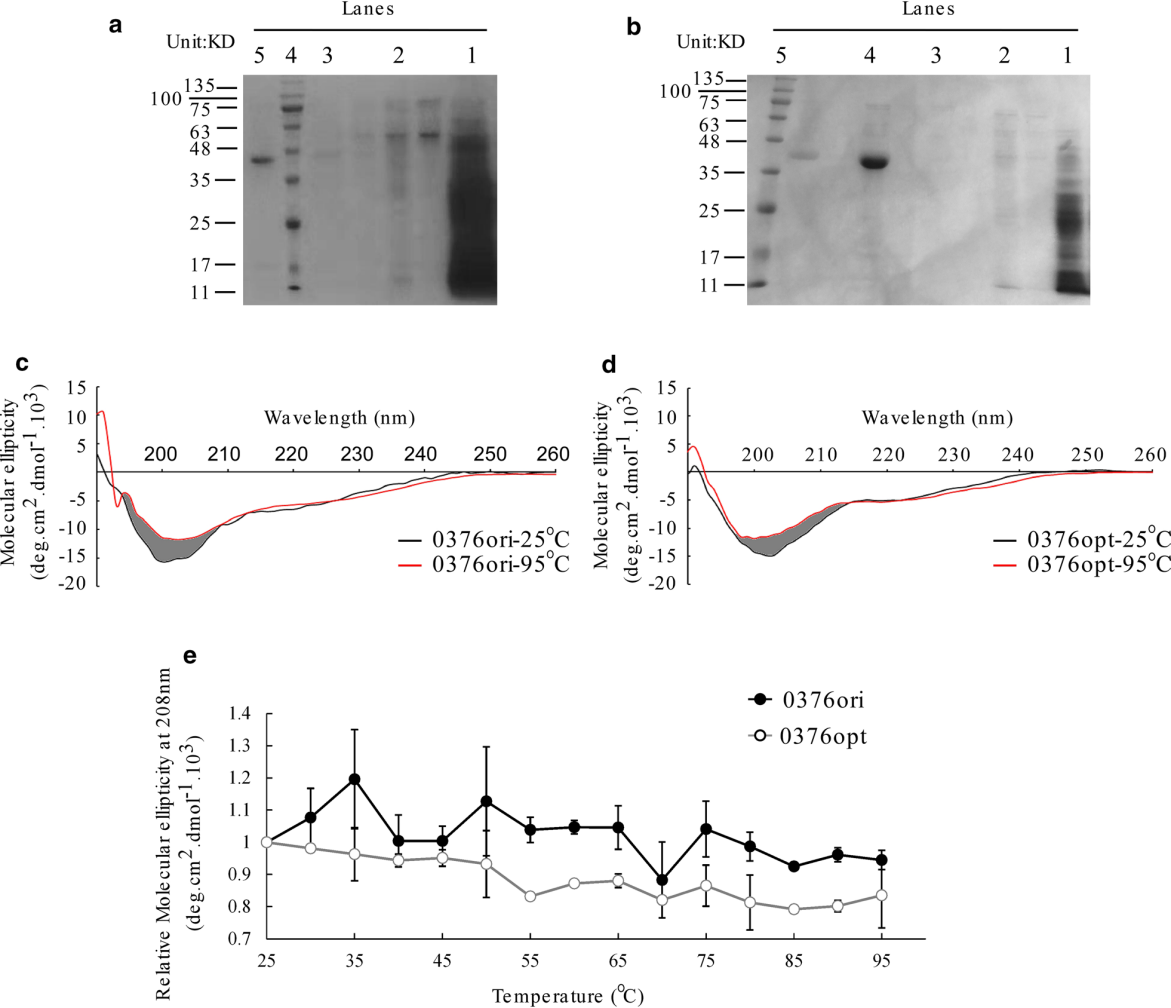
Circular Dichroism (CD) spectra of 0376ori and 0376opt proteins under different temperatures. **a**, **b** Protein purification of 0376ori (**a**) and 0376opt (**b**) by affinity chromatography. In **a**: lane 1, flow through; lane 2, wash with 10 mM imidazole; lane 3, wash with 20 mM imidazole; lane 4, marker; lane 5, elution with 150 mM imidazole. In **b**: lane 1, flow through; lane 2, wash with 10 mM imidazole; lane 3, wash with 20 mM imidazole; lane 4, elution with 150 mM imidazole; lane 5, marker. Unlabeled lanes are irrelevant to this experiment. **c**, **d** CD spectra of purified 0376ori (**c**) and 0376opt (**d**) proteins under 25 °C and 95 °C. The difference in between is shadowed by grey. **e** Temperature mediated molecular ellipticity (208 nm) decay of 0376ori and 0376opt. Error bars were calculated as standard deviation of three independent experiments

Altered stabilities towards trypsin digestion and freeze–thaw cycles suggested a conformation change. We then performed Circular Dichroism (CD) spectroscopy on purified proteins to directly reveal any conformation difference. Transgenic strains expressing original or codon optimized *0376* sequences under the strong *AOX1* promoter were constructed. Secretive expression was tried first for easier protein purification. However, proteins were easily degraded after secretion. We then performed intracellular protein expression and purification. A 6 × His tag was fused at C-terminus of 0376ori and 0376opt proteins and purified by affinity chromatography (Fig. 6a, b). Proteins were then scanned for CD spectra at different temperatures from 25 to 95 °C. Interestingly, both 0376ori and 0376opt showed stable secondary structures at high temperature since significant ellipticity could still be detected at 95 °C (Fig. 6c, d). Although their ellipticity curves at 25 °C are comparable, a different shift could still be observed with increased temperatures (Fig. 6c, d and Additional file 1: Figure S3). The decreased ellipticity was mostly located within 195–205 nm wavelength region for 0376ori, and 200–210 nm wavelength region for 0376opt (Fig. 6c, d, grey region). Since θ_208_ was a typical marker for α-helix [41], we then compared the ellipticity decay rates along temperature. As shown by Fig. 6e, θ_280_ of 0376opt decayed to around 80% at high temperatures (above 85 °C), while that of 0376ori appeared more stable.

**Fig. 7:**
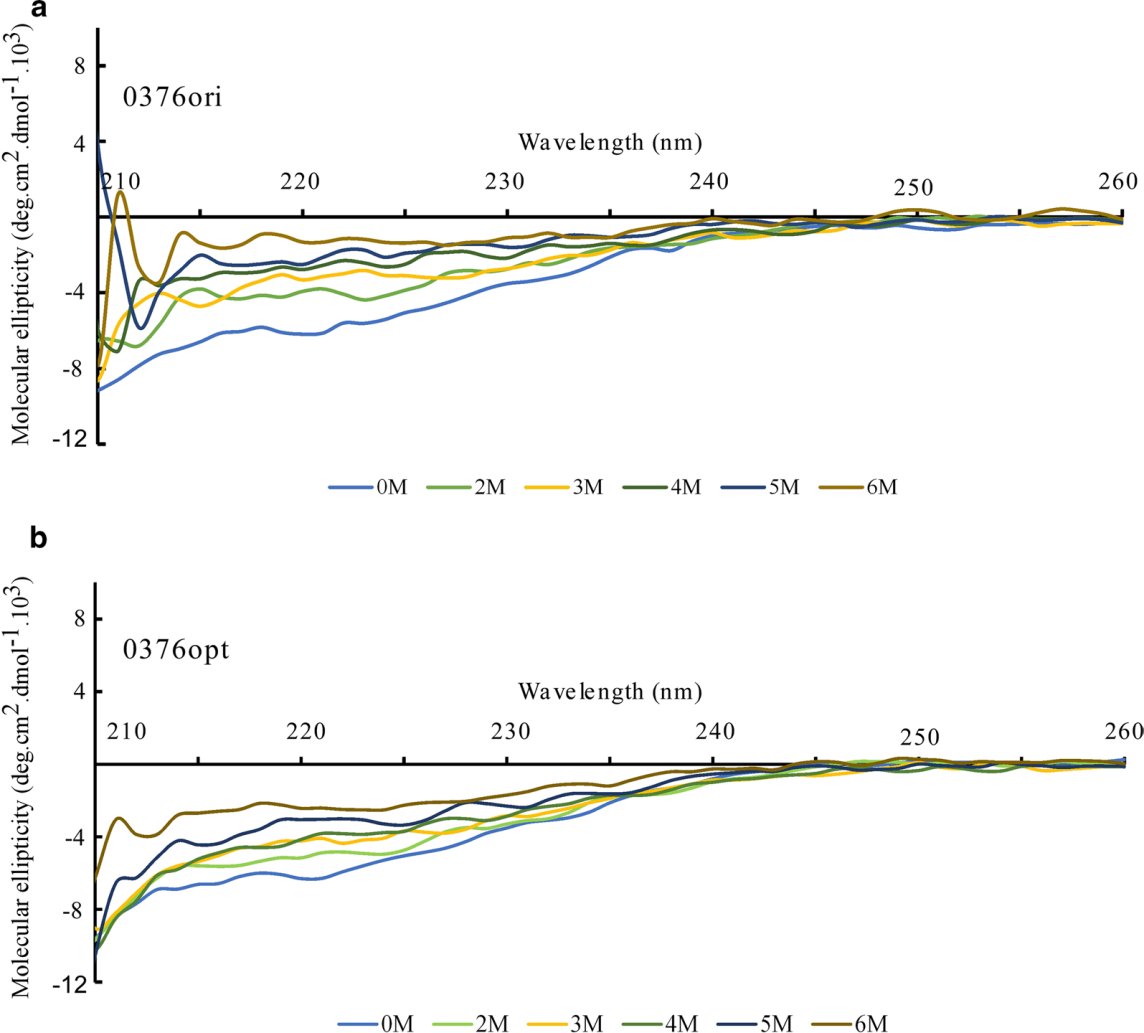
Circular Dichroism (CD) spectra of 0376ori (**a**) and 0376opt (**b**) proteins with urea addition. Urea concentrations used in each curve are indicated at the bottom

Since the secondary structures in both 0376ori and 0376opt are quite stable with temperature change, urea treatment was then tried to compare their sensitivities. Urea is known to denature proteins by interacting with both nonpolar and polar components [42]. Ellipticity plots started from 210 nm, since urea addition created huge and random oscillations before that. Interestingly, ellipticity was decreased to half starting with 3-4 M urea on 0376ori (Fig. 7a), while 6 M urea was needed to achieve a similar phenotype on 0376opt (Fig. 7b). Taken together, CD spectroscopy on 0376ori and 0376opt proteins suggests that although the ellipticity curves under room temperature are comparable, their conformations respond differentially upon increased temperature or urea addition.

## Discussion

*Pichia pastoris* is a strong system which is heavily used for recombinant protein expression. Its efficiency is tightly correlated with any regulatory mechanisms during protein anabolism. Here we examined the codon bias profile in *P. pastoris* genome first, and found a direct correlation between codon translation efficiency and usage frequency. A narrow N-terminal ramp with rare codons was also identified in *P. pastoris* genome, being stronger in secreted proteins. However, represented by several examples, the secretion ability of N-terminal signal peptides was tolerant towards synonymous codon changes. We then picked two gene candidates with different level of structural disorder and studied how their expression profiles were affected by full-length codon optimization. Finally, protein conformation change brought by codon optimization was confirmed by protein stability assays and CD spectroscopy.

Although the codon choice of N-terminal signal peptides does not affect their secretion ability significantly, it did regulate protein expression levels. And the level change seems to be proportional with the length optimized or de-optimized region, in which the longer α-mating factor prepro-peptide had a larger impact than those shorter SPs. The 5′ “ramp” composed by rare codons has been reported to be beneficial for protein synthesis in many other organisms [4, 43]. It is likely that its significant role was masked by the secretion process itself, or by the “special” codon bias of *P. pastoris* genome (a preference to use A/U in frequent codons).

Suggested by the trypsin sensitivity assay, freeze–thaw assay and CD spectroscopy, codon optimization may also alter protein conformation even though expression has been successfully kept. Combined with our pervious study [6, 10], structurally disordered proteins are peculiarly prone to be affected. Here this effect was confirmed in *P. pastoris* with more structural evidence. Interestingly, the 0376ori and 0376opt proteins we tested here showed different phenotypes in different stability assays (summarized by Table 2). However, this may not be contradictory since each assay is testing a specific structural feature. Trypsin sensitivity is determined by putative lysine and arginine residues at the protein surface, which may be altered by codon optimization. Both freeze–thaw [44] and urea [42] start with damaging surface structures, in which 0376opt looks more stable. Temperature increase brings an overall challenge to secondary bonds stabilizing protein structure. Anyway, here we suggest that extreme codon optimization may not be appropriate for structurally disordered proteins, and this is also a potential check point for problem solving in those failed examples.

**Table 2:** Stability comparison between 0376ori and 0376opt proteins in different assays

	0376ori	0376opt
Stability under trypsin treatment	More stable	Less stable
Freeze–thaw stability	Less stable	More stable
Temperature mediated denaturation of θ_208_	More stable	Less stable
Urea mediated denaturation	Less stable	More stable

Different genes seem to have different performances during codon optimization, represented by success rate, expression level and activity. This difference is perfectly displayed in the two gene candidates picked here. Each protein coding gene has a distinct sequence which will result in different GC content and amino acid combination. Therefore, optimal codon choice may create certain sequence or structural features to affect transcriptional and translational events. The possible conflict between natural design and manual manipulation may be the most challenging part when designing codon optimization strategies.

Taken together, codon bias is an important factor regulating protein expression in *P. pastoris*. For some genes, an “updated” codon optimization strategy should be adopted to ensure appropriate expression and conformation. In this “updated” strategy, more aspects including protein structure and transcription are considered on the basis of traditional codon optimization method.

## Supplementary Information

**Additional file 1: Figure S1.** CAI plots of the 39 endogenous secretory SPs in *P. pastoris*. **Figure S2.** CAI, extracellular & intracellular enzyme activity of amylase reporter, as well as secretion ability of SP15 (A), SP20 (B), SP23 (C) and SP34 (D) when codons were de-optimized or optimized. **Figure S3.** Circular Dichroism (CD) spectra of 0376ori and 0376opt proteins under increasing temperatures.** Table S1.** Relative codon usage frequency (ω) in *P. pastoris *(synonymous codons with highest usage frequency are set as 1). **Table S2.** tAI values of *P. pastoris *codons. **Table S3.** Codon optimization and de-optimization sequences of secretive signals. **Table S4.** *0376 *and *0432 *sequences before and after codon optimization.

## Data Availability

All data generated or analyzed during this study are included in this published article [and its additional information files]. Materials used in this study are available from the corresponding author on reasonable request.

## References

[1] Sharp PM, Li WH (1986). Codon usage in regulatory genes in *Escherichia coli* does not reflect selection for ‘rare’ codons. Nucleic Acids Res.

[2] Ikemura T (1981). Correlation between the abundance of *Escherichia coli* transfer RNAs and the occurrence of the respective codons in its protein genes. J Mol Biol.

[3] Krüger MK, Sørensen MA (1998). Aminoacylation of hypomodified tRNA Glu in vivo. J Mol Biol.

[4] Tuller T, Carmi A, Vestsigian K, Navon S, Dorfan Y, Zaborske J (2010). An Evolutionarily conserved mechanism for controlling the efficiency of protein translation. Cell.

[5] Drummond AD, Wilke CO (2008). Mistranslation-induced protein misfolding as a dominant constraint on coding-sequence evolution. Cell.

[6] Zhou M, Guo J, Cha J, Chae M, Chen S, Barral JM (2013). Non-optimal codon usage affects expression, structure and function of clock protein FRQ. Nature.

[7] Fu J, Murphy KA, Zhou M, Li YH, Lam VH, Tabuloc CA (2016). Codon usage affects the structure and function of the Drosophila circadian clock protein PERIOD. Genes Dev.

[8] Xu Y, Ma P, Shah P, Rokas A, Liu Y, Johnson CH (2013). Non-optimal codon usage is a mechanism to achieve circadian clock conditionality. Nature.

[9] Zhou Z, Dang Y, Zhou M, Yuan H, Liu Y (2018). Codon usage biases co-evolve with transcription termination machinery to suppress premature cleavage and polyadenylation. Elife.

[10] Zhou M, Wang T, Fu J, Xiao G, Liu Y (2015). Nonoptimal codon usage influences protein structure in intrinsically disordered regions. Mol Microbiol.

[11] Zhou Z, Dang Y, Zhou M, Li L, Yu CH, Fu J (2016). Codon usage is an important determinant of gene expression levels largely through its effects on transcription. Proc Natl Acad Sci USA.

[12] Shi Y, Manley JL (2015). The end of the message: multiple protein–RNA interactions define the mRNA polyadenylation site. Genes Dev.

[13] Goodman DB, Church GM, Kosuri S (2013). Causes and effects of N-terminal codon bias in bacterial genes. Science.

[14] Blobel G, Dobberstein B (1975). Transfer of proteins across membranes. I. Presence of proteolytically processed and unprocessed nascent immunoglobulin light chains on membrane-bound ribosomes of murine myeloma. J Cell Biol.

[15] Rapoport TA (2007). Protein translocation across the eukaryotic endoplasmic reticulum and bacterial plasma membranes. Nature.

[16] Liu H, Rahman SU, Mao Y, Xu X, Tao S (2017). Codon usage bias in 5′ terminal coding sequences reveals distinct enrichment of gene functions. Genomics.

[17] Zalucki YM, Beacham IR, Jennings MP (2009). Biased codon usage in signal peptides: a role in protein export. Trends Microbiol.

[18] Power PM, Jones RA, Beacham IR, Bucholtz C, Jennings MP (2004). Whole genome analysis reveals a high incidence of non-optimal codons in secretory signal sequences of *Escherichia coli*. Biochem Biophys Res Commun.

[19] Zalucki YM, Jennings MP (2007). Experimental confirmation of a key role for non-optimal codons in protein export. Biochem Biophys Res Commun.

[20] Zalucki YM, Gittins KL, Jennings MP (2008). Secretory signal sequence non-optimal codons are required for expression and export of beta-lactamase. Biochem Biophys Res Commun.

[21] Huang M, Gao Y, Zhou X, Zhang Y, Cai M (2017). Regulating unfolded protein response activator HAC1p for production of thermostable raw-starch hydrolyzing Α-amylase in *Pichia pastoris*. Bioprocess Biosyst Eng.

[22] Krainer FW, Gerstmann MA, Darnhofer B, Birner-Gruenberger R, Glieder A (2016). Biotechnological advances towards an enhanced peroxidase production in *Pichia pastoris*. J Biotechnol.

[23] Wang X, Wang Q, Wang J, Bai P, Shi L, Shen W (2016). Mit1 transcription factor mediates methanol signaling and regulates the alcohol oxidase 1 (AOX1) promoter in *pichia pastoris*. J Biol Chem.

[24] Nordén K, Agemark M, Danielson JÅ, Alexandersson E, Kjellbom P, Johanson U (2011). Increasing gene dosage greatly enhances recombinant expression of aquaporins in *Pichia pastoris*. BMC Biotechnol.

[25] Wang J, Wang X, Shi L, Qi F, Zhang P, Zhang Y (2017). Methanol-independent protein expression by AOX1 promoter with trans-acting elements engineering and glucose-glycerol-shift induction in *Pichia pastoris*. Sci Rep.

[26] Xu N, Zhu J, Zhu Q, Xing Y, Cai M, Jiang T (2018). Identification and characterization of novel promoters for recombinant protein production in yeast *Pichia pastoris*. Yeast.

[27] Navone L, Vogl T, Luangthongkam P, Blinco JA, Luna-Flores C, Chen X (2021). Synergistic optimisation of expression, folding, and secretion improves *E. coli* AppA phytase production in *Pichia pastoris*. Microb Cell Fact.

[28] Demir I, Calik P (2020). Hybrid-architectured double-promoter expression systems enhance and upregulate-deregulated gene expressions in *Pichia pastoris* in methanol-free media. Appl Microbiol Biotechnol.

[29] Xu Q, Bai C, Liu Y, Song L, Tian L, Yan Y (2019). Modulation of acetate utilization in *Komagataella phaffii* by metabolic engineering of tolerance and metabolism. Biotechnol Biofuels.

[30] Liu Y, Tu X, Xu Q, Bai C, Kong C, Liu Q (2018). Engineered monoculture and co-culture of methylotrophic yeast for de novo production of monacolin J and lovastatin from methanol. Metab Eng.

[31] Schutter KD, Lin YC, Tiels P, Hecke AV, Glinka S, Weber-Lehmann J (2009). Genome sequence of the recombinant protein production host *Pichia pastoris*. Nat Biotechnol.

[32] Outchkourov NS, Stiekema WJ, Jongsma MA (2002). Optimization of the expression of equistatin in *Pichia pastoris*. Protein Expr Purif.

[33] Hu S, Li L, Qiao J, Guo Y, Cheng L, Liu J (2006). Codon optimization, expression, and characterization of an internalizing anti-ErbB2 single-chain antibody in *Pichia pastoris*. Protein Expr Purif.

[34] Zhao S, Huang J, Zhang C, Deng L, Hu N, Liang Y (2010). High-level expression of an *Aspergillus niger* endo-beta-1,4-glucanase in *Pichia pastoris* through gene codon optimization and synthesis. J Microbiol Biotechnol.

[35] Lincereghino GP, Stark CM, Kim D, Chang J, Shaheen N, Poerwanto H (2013). The effect of α-mating factor secretion signal mutations on recombinant protein expression in *Pichia pastoris*. Gene.

[36] Pechmann S, Frydman J (2013). Evolutionary conservation of codon optimality reveals hidden signatures of co-translational folding. Nat Struct Mol Biol.

[37] Reis MD, Savva R, Wernisch L (2004). Solving the riddle of codon usage preferences: a test for translational selection. Nucleic Acids Res.

[38] Sharp PM, Li WH (1987). The codon adaptation index—a measure of directional synonymous codon usage bias, and its potential applications. Nucleic Acids Res.

[39] Huang CJ, Damasceno LM, Anderson KA, Zhang S, Old LJ, Batt CA (2011). A proteomic analysis of the *Pichia pastoris* secretome in methanol-induced cultures. Appl Microbiol Biotechnol.

[40] Wang Y, Mao Y, Xu X, Tao S, Chen H (2015). Codon usage in signal sequences affects protein expression and secretion using baculovirus/insect cell expression system. PLoS ONE.

[41] Greenfield NJ (2006). Using circular dichroism spectra to estimate protein secondary structure. Nat Protoc.

[42] Rossky P (2008). Protein denatureation by urea: Slash and bond. Proc Natl Acad Sci USA.

[43] Verma M, Choi J, Cottrell KA, Lavaqniono Z, Thomas EN, Pavlovic-Djuranovic S (2019). A short translational ramp determines the efficiency of protein synthesis. Nat Commun.

[44] Cao E, Chen Y, Cui Z, Foster P (2002). Effect of freezing and thawing rates on denaturation of proteins in aqueous solutions. Biotechnol Bioeng.

