# Supplementary material for: Codon usage bias regulates gene expression and protein conformation in yeast expression system P. pastoris
Source: Microb Cell Fact. 2021 Apr 26;20:91. doi: 10.1186/s12934-021-01580-9 (PMC8077831; doi:10.1186/s12934-021-01580-9)
Supplement: Supplementary file 1 — Additional file 1: Figure S1. CAI plots of the 39 endogenous secretory SPs in P. pastoris. Figure S2. CAI, extracellular & intracellular enzyme activity of amylase reporter, as well as secretion ability of SP15 (A), SP20 (B), SP23 (C) and SP34 (D) when codons were de-optimized or optimized. Figure S3. Circular Dichroism (CD) spectra of 0376ori and 0376opt proteins under increasing temperatures. Table S1. Relative codon usage frequency (ω) in P. pastoris (synonymous codons with highest usage frequency are set as 1). Table S2. tAI values of P. pastoris codons. Table S3. Codon optimization and de-optimization sequences of secretive signals. Table S4. 0376 and 0432 sequences before and after codon optimization. [file 12934_2021_1580_MOESM1_ESM.pdf]

Table S1. Relative codon usage frequency (ω) in *P. pastoris* (synonymous codons with highest usage frequency are set as 1)

| | | | | | |
|---|---|---|---|---|---|
| Phe | UUU | 1.000 | Ser | UCU | 1.000 |
| | UUC | 0.855 | | UCC | 0.676 |
| Leu | UUA | 0.495 | | UCA | 0.623 |
| | UUG | 1.000 | | UCG | 0.303 |
| | CUU | 0.505 | Pro | CCU | 0.836 |
| | CUC | 0.241 | | CCC | 0.360 |
| | CUA | 0.340 | | CCA | 1.000 |
| | CUG | 0.473 | | CCG | 0.206 |
| Ile | AUU | 1.000 | Thr | ACU | 1.000 |
| | AUC | 0.624 | | ACC | 0.647 |
| | AUA | 0.357 | | ACA | 0.616 |
| Met | AUG | 1.000 | | ACG | 0.268 |
| Val | GUU | 1.000 | Ala | GCU | 1.000 |
| | GUC | 0.554 | | GCC | 0.574 |
| | GUA | 0.369 | | GCA | 0.522 |
| | GUG | 0.457 | | GCG | 0.135 |
| Tyr | UAU | 0.884 | Cys | UGU | 1.000 |
| | UAC | 1.000 | | UGC | 0.571 |
| Tem | UAA | 1.000 | Tem | UGA | 0.375 |
| | UAG | 0.625 | Trp | UGG | 1.000 |
| His | CAU | 1.000 | Arg | CGU | 0.343 |
| | CAC | 0.771 | | CGC | 0.109 |
| Gln | CAA | 1.000 | | CGA | 0.209 |
| | CAG | 0.642 | | CGG | 0.095 |
| Asn | AAU | 0.940 | Ser | AGU | 0.512 |
| | AAC | 1.000 | | AGC | 0.311 |
| Lys | AAA | 0.885 | Arg | AGA | 1.000 |
| | AAG | 1.000 | | AGG | 0.328 |
| Asp | GAU | 1.000 | Gly | GGU | 1.000 |
| | GAC | 0.725 | | GGC | 0.318 |
| Glu | GAA | 1.000 | | GGA | 0.749 |
| | GAG | 0.775 | | GGG | 0.227 |

Table S2. tAI values of *P. pastoris* codons

| | | | | | |
|---|---|---|---|---|---|
| Phe | UUU | 0.4917 | Ser | UCU | 0.6667 |
| | UUC | 0.8333 | | UCC | 0.2733 |
| Leu | UUA | 0.3867 | | UCA | 0.48 |
| | UUG | 0.5533 | | UCG | 0.3333 |
| | CUU | 0.1667 | Pro | CCU | 0.38 |
| | CUC | 0.1667 | | CCC | 0.24 |
| | CUA | 0.24 | | CCA | 0.3333 |
| | CUG | 0.3333 | | CCG | 0.6667 |
| Ile | AUU | 0.8333 | Thr | ACU | 0.8333 |
| | AUC | 0.6 | | ACC | 0.22 |
| | AUA | 0.1668 | | ACA | 0.6 |
| Met | AUG | 0.616 | | ACG | 0.1668 |
| Val | GUU | 0.8333 | Ala | GCU | 0.8333 |
| | GUC | 0.22 | | GCC | 0.1067 |
| | GUA | 0.1668 | | GCA | 0.6 |
| | GUG | 0.6 | | GCG | 0.3334 |
| Tyr | UAU | 0.6667 | Cys | UGU | 0.1967 |
| | UAC | 0.3933 | | UGC | 0.3333 |
| Tem | UAA | / | Tem | UGA | / |
| | UAG | / | Trp | UGG | 0.5 |
| His | CAU | 0.295 | Arg | CGU | 0.22 |
| | CAC | 0.5 | | CGC | 0.38 |
| Gln | CAA | 0.4933 | | CGA | 0.1667 |
| | CAG | 0.5 | | CGG | 0.6667 |
| Asn | AAU | 0.6667 | Ser | AGU | 0.3334 |
| | AAC | 0.3933 | | AGC | 0.1967 |
| Lys | AAA | 0.5 | Arg | AGA | 0.3333 |
| | AAG | 0.9933 | | AGG | 0.24 |
| Asp | GAU | 0.59 | Gly | GGU | 0.4917 |
| | GAC | 1 | | GGC | 0.8333 |
| Glu | GAA | 0.9333 | | GGA | 0.3267 |
| | GAG | 0.8333 | | GGG | 0.5 |

Table S3 Codon optimization and de-optimization sequences of secretive signals

| | Original | | Optimization | | De-optimization | | N-terminal optimization | | C-terminal optimization | |
|---|---|---|---|---|---|---|---|---|---|---|
| | Sequence | CAI | Sequence | CAI | Sequence | CAI | Sequence | CAI | Sequence | CAI |
| sp 9 | ATGAAATTGT<br>TGAACTTTCT<br>GCTTAGCTTC<br>GTAACTCTGT<br>TCGGACTATT<br>ATCAGGTTCT<br>GTGTTTGCA | 0.683 | ATGAAGTTGT<br>TGAACTTTTT<br>GTTGTCTTTT<br>GTTACTTTGT<br>TTGGTTTGTT<br>GTCTGGTTCT<br>GTTTTTGCT | 1 | ATGAAACTCC<br>TCAATTTCCT<br>CCTCTCGTTC<br>GTAACGCTCT<br>TCGGGCTCCT<br>CTCGGGGTCG<br>GTATTCGCG | 0.374 | / | | / | |
| sp 20 | ATGAGCACCC<br>TGACATTGCT<br>GGCTGTGCTG<br>TTGTCGCTTC<br>AAAATTCAGC<br>TCTTGCT | 0.649 | ATGTCTACTT<br>TGACTTTGTT<br>GGCTGTTTTG<br>TTGTCTTTGC<br>AAAACTCTGC<br>TTTGGCT | 1 | ATGTCGACGC<br>TCACGCTCCT<br>CGCGGTACTC<br>CTCTCGCTCC<br>AGAATTCGGC<br>GCTCGCG | 0.287 | / | | / | |
| sp 13 | ATGCTATCAA<br>CTATCTTAAA<br>TATCTTTATC<br>CTGTTGCTCT<br>TCATACAGGC<br>ATCCCTACAG | 0.604 | ATGTTGTCTA<br>CTATTTTGAA<br>CATTTTTATTT<br>TGTTGTTGTTT<br>ATTCAAGCTT<br>CTTTGCAA | 1 | ATGCTCTCGA<br>CGATACTCAA<br>TATATTCATA<br>CTCCTCCTCT<br>TCATACAGGC<br>GTCGCTCCAG | 0.375 | / | | / | |
| sp 15 | ATGTTTAAAT<br>CTCTGTGCAT<br>GTTAATAGGA<br>TCCTGCCTAT<br>TGAGTTCAGT<br>CTTGGCA | 0.661 | ATGTTTAAGT<br>CTTTGTGTAT<br>GTTGATTGGT<br>TCTTGTTTGTT<br>GTCTTCTGTTT<br>TGGCT | 1 | ATGTTCAAAT<br>CGCTCTGCAT<br>GCTCATAGGG<br>TCGTGCCTCC<br>TCTCGTCGGT<br>ACTCGCG | 0.372 | / | | / | |
| sp 23 | ATGAAAATAT<br>TAAGTGCATT<br>GCTTCTTCTTT<br>TTACGTTGGC<br>CTTTGCT | 0.642 | ATGAAGATTT<br>TGTCTGCTTT<br>GTTGTTGTTG<br>TTTACTTTGG<br>CTTTTGCT | 1 | ATGAAAATAC<br>TCTCGGCGCT<br>CCTCCTCCTC<br>TTCACGCTCG<br>CGTTCGCG | 0.314 | / | | / | |
| sp 34 | ATGAGACCAG<br>TGCTTTCGTT<br>ATTACTCTTG<br>CTGGCTTCTT<br>CGGTACTCGC<br>T | 0.560 | ATGAGACCAG<br>TTTTGTCTTTG<br>TTGTTGTTGTT<br>GGCTTCTTCT<br>GTTTTGGCT | 1 | ATGCGCCCGG<br>TACTCTCGCT<br>CCTCCTCCTC<br>CTCGCGTCGT<br>CGGTACTCGC<br>G | 0.253 | / | | / | |
| α-factor | ATGAGATTTC<br>CTTCAATTTTT<br>ACTGCAGTTT<br>TATTCGCAGC<br>ATCCTCCGCA<br>TTAGCTGCTC<br>CAGTCAACAC<br>TACAACAGAA<br>GATGAAACG<br>GCACAAATTC<br>CGGCTGAAGC<br>TGTCATCGGT<br>TACTCAGATT<br>TAGAAGGGG<br>ATTTCGATGT<br>TGCTGTTTTG<br>CCATTTTCCA<br>ACAGCACAA<br>ATAACGGGTT<br>ATTGTTTATA<br>AATACTACTA<br>TTGCCAGCAT<br>TGCTGCTAAA<br>GAAGAAGGG<br>GTATCTCTCG<br>AGAAAAGAG<br>AGGCTGAAGC<br>TTAC | 0.746 | ATGAGATTTC<br>CATCTATTTTT<br>ACTGCTGTTT<br>TGTTTGCTGC<br>TTCTTCTGCTT<br>TGGCTGCTCC<br>AGTCAACACT<br>ACAACAGAA<br>GATGAAACTG<br>CTCAAATTCC<br>AGCTGAAGCT<br>GTTATTGGTT<br>ACTCTGATTT<br>GGAAGGTGAT<br>TTTGATGTTG<br>CTGTTTTGCC<br>ATTTTCTAAC<br>TCTACTAACA<br>ACGGTTTGTT<br>GTTTATTAAC<br>ACTACTATTG<br>CTTCTATTGC<br>TGCTAAGGAA<br>GAAGGTGTTT<br>CTTTGGAAAA<br>GAGAGAAGC<br>TGAAGCTTAC | 0.983 | ATGCGGTTCC<br>CGTCGATATT<br>CACGGCGGTA<br>CTCTTCGCGG<br>CGTCGTCGGC<br>GCTCGCGGCG<br>CCGGTAAATA<br>CGACGACGG<br>AGGACGAGA<br>CGGCGCAGAT<br>ACCGGCGGA<br>GGCGGTAATA<br>GGGTATTCGG<br>ACCTCGAGGG<br>GGACTTCGAC<br>GTAGCGGTAC<br>TCCCGTTCTC<br>GAATTCGACG<br>AATAATGGGC<br>TCCTCTTCAT<br>AAATACGACG<br>ATAGCGTCGA<br>TAGCGGCGAA<br>AGAGGAGGG<br>GGTATCGCTC<br>GAGAAACGG<br>GAGGCGGAG<br>GCGTAT | 0.348 | ATGAGATTTC<br>CATCTATTTTT<br>ACTGCTGTTT<br>TGTTTGCTGC<br>TTCTTCTGCTT<br>TGGCTGCTCC<br>AGTCAACACT<br>ACAACAGAA<br>GATGAAACGG<br>CACAAATTCC<br>GGCTGAAGCT<br>GTCATCGGTT<br>ACTCAGATTT<br>AGAAGGGGA<br>TTTCGATGTT<br>GCTGTTTTGC<br>CATTTTCCAA<br>CAGCACAAAT<br>AACGGGTTAT<br>TGTTTATAAA<br>TACTACTATT<br>GCCAGCATTG<br>CTGCTAAAGA<br>AGAAGGGGT<br>ATCTCTCGAG<br>AAAAGAGAG<br>GCTGAAGCTT<br>AC | 0.794 | ATGAGATTTC<br>CTTCAATTTTT<br>ACTGCAGTTT<br>TATTCGCAGC<br>ATCCTCCGCA<br>TTAGCTGCTC<br>CAGTCAACAC<br>TACAACAGAA<br>GATGAAACTG<br>CTCAAATTCC<br>AGCTGAAGCT<br>GTTATTGGTT<br>ACTCTGATTT<br>GGAAGGTGAT<br>TTTGATGTTG<br>CTGTTTTGCC<br>ATTTTCTAAC<br>TCTACTAACA<br>ACGGTTTGTT<br>GTTTATTAAC<br>ACTACTATTG<br>CTTCTATTGC<br>TGCTAAGGAA<br>GAAGGTGTTT<br>CTTTGGAAAA<br>GAGAGAAGC<br>TGAAGCTTAC | 0.923 |

Table S4 *0233*, *0376* and *0432* sequences before and after codon optimization.

| | |
|---|---|
| 0376 | ATGAAGAGCTGGCAGTTTGATGAGGAAATAGCTCTTCTCCGTGCAGCATGTTACAATCGTCCTGTAGGAAAGGACAAAGCCAAGCATTTGGAGGCTTTGGTAAAGGTTTTATCTGATGCTGGGTATGCAGTAACCGAAAAGGATGCCTGGGATAAGTTAGCGGAGTACTACGATTTCAAGCAATTGGAAAACTTGGATCTATCCGACCAGTCTCCAAATGAAGAGGACATTGCTTCAAGAGATGTGTCCAATGACGAGGCACAAAACGAAGACGAGACTGATGCCACCGGTGTTGGCGAACCCGAGCACATGTTAGAACGAGAAGGAACACCAAGAACTCGAACAAGATCAACTAAGATTGAAAAAACTCTCACACCAACAACCAAGGGCCGTAAGCGAATAGATAATTCAGACGAATCTGACAGATCGGATAGGGAGCGGAGTAGAACGCCCGTCAATAAAGGAATTGGATATAGAACAAGACGGAGAACTCCCAAAGACCGTCTAAAAGATCAAGATGATGTTGACGGCAGCAGTAGAAACTCTAATGACGAAGACACGAGGAACAACGATAGTGAGCTAGATGATACAGATACACCCAAAAGAGTCACTAGAGCGTCTTCTAGAATATCTAGAAGAACTCCACGCAAGGACAAAGACACCATTAAAGACCCCAAAGATACAACGAGCAAGAGGCGAACTCGGGCAAGGTGA |
| 0376 opt | ATGAAATCTTGGCAATTTGATGAAGAAATTGCTTTGTTGAGAGCTGCTTGTTATAATAGACCAGTTGGAAAAGATAAAGCTAAACATTTGGAAGCTTTGGTTAAAGTTTTGTCTGATGCTGGATATGCTGTTACTGAAAAAGATGCTTGGGATAAATTGGCTGAATATTATGATTTTAAACAATTGGAAAATTTGGATTTGTCTGATCAATCTCCAAATGAAGAAGATATTGCTTCTAGAGATGTTTCTAATGATGAAGCTCAAAATGAAGATGAAACTGATGCTACTGGAGTTGGAGAACCAGAACATATGTTGGAAAGAGAAGGAACTCCAAGAACTAGAACTAGATCTACTAAAATTGAAAAAACTTTGACTCCAACTACTAAAGGAAGAAAAAGAATTGATAATTCTGATGAATCTGATAGATCTGATAGAGAAAGATCTAGAACTCCAGTTAATAAAGGAATTGGATATAGAACTAGAAGAAGAACTCCAAAAGATAGATTGAAAGATCAAGATGATGTTGATGGATCTTCTAGAAATTCTAATGATGAAGATACTAGAAATAATGATTCTGAATTGGATGATACTGATACTCCAAAAAGAGTTACTAGAGCTTCTTCTAGAATTTCTAGAAGAACTCCAAGAAAAGATAAAGATACTATTAAAGATCCAAAAGATACTACTTCTAAAAGAAGAACTAGAGCTAGATGA |
| 0432 | ATGTCCAAAACAAATTCGACGGTAAAAGGAAGGCCCAAACTAGTTCAATCTGTGAACGGATTTTTTTCCAAAGGTGCGAATGTGGTTACACACGGACGATTTTTTTCATCCGAACCTTCTAAAAGTGAAGATTCAGGTCTGGATTTTGTTAAACCCAATAAATGGTTCAACTTACTGGTTCCAGAACTGTCCTCTAGTGCTAAGATTGCATTATCTCGTTGGAAAGGTAATGACCTACTAACGATCCCACCCATGGTTTTAGAAGTTTTTCTGGTAGTTCCAACAGAGCATTCAGATAGTCTGGTCCTTCGTGGGGGAGCCGATGGATTGAGCCAAGATGATGTCGTCATCAACGTTGGTAACTACGAGCGCAACGAGATTGTTCTAGAACGATGGTTACTAGAATTTGATTTGACTACCCTGGACAAAAATTCTACTGAGATCTACGGTATTTACAAGAAAATGATCATTCTTTTTCGCAATCTCTACACATTTGTACGATTGATGCCTGCATTTAGGCTCTTTCAAGAGGGGAACTGGAAAATAGGGACTCGAACATTAGATGGTAGCGAGCCAATCTCCAGCAAAGATCGAATTGGTTTAAGTGATTCGTTCCTGGGCGAATCGAAGAATTCAGAAGAGCAAGATCAAAGTCAGTGCTATTATTCCCATTTGAGCCAAAAAAAATTTCGATCTATAACCACTTCAATGGGTAGTTTGAAGATCAGTTGCAGCTTCCGTAGAAATACTGCCTTTCGGTGGGCAAGCACACGTCGAAAAGCGCGTGATAACACGGTCACAGGTGGGCTTTCTCGGGCTGTTGGAATACAACCTTTCAAGACTGGTGTTTTATCGTCCAGCCCTGGTCGTAGTCCCGGTCATGGCTTCACTGCGACACTTTGTGCCCGGACTGAGTCACAGCCTATCCCTCTTCAAATTCATCATCGCTCAAGCTCTAACGCTTCCCTTGTACAACTTCTTCGGAATCCAAGAAATAGCATTCCAACGTCAATAAACTCCGTGCTTGAGGACCATTCAACAATCTCTCCAGCAACAAAATTCTCTTCCTCTTTTAGGTTAGCACGCAGAGGGAGTCTCCATTCCCGAACATCGGTCGATCGTCGTTCAAGTGACTTATCATCGTCTGACATAGACCCAGACCAATTCTACGTCGATGAAGATATAAATGACCTCATGCGTATGATTGATGCCAGGCCGAATTTAAGGCTTTCCTCAGCACGGTCACGTGAAACTTCACCGTCATCTCTGAATCGATTTCAACTCTTACAGAAAACTCATGATATCTTGAGTGATTCGGTACATGCAAGTTTACCCACAAGTGCCCATGGAGTTCTCTCATTGTCCCCATCACGAAGGTATTCCATATCACCTCCAACCATGGGGCCAGGCTCATCTACAGCCTCAATCTCTCAATCTTTAACTTACGCACGAATGGATCACCAGGACTCCGCACAATCAGCCAATTCTATCCGT |

| | |
|---|---|
| | GACATTTTGCAGAGTTCGAGCCGACGTAACTCTTCTTCCAACAGACGAGGATCTGGCCAGTCGCC<br>AAGGCCTGGAACAATACTAGGACTACCATCCGGGCTAGGCTCTGGTGACAGTGCGATATCTGAT<br>GAGGCTTCTAAAGAACATGTTTATGAAGAGCACGCAATAATTGATGATGATGAGGAGGAGGAA<br>GACATGATCACTCAAAGAAATTTGAATCATCTATTGAAGTACAGAAAACTTCGCCTTCAAGATAC<br>GAAAAAAAGCAAAGAGATCACGATCAAAGAAGAGGATGACGATTTGTTGTTCACAATGAGCGA<br>CATGAATCTTAGCTGA |
| 0432opt | ATGTCTAAAACTAATTCTACTGTTAAAGGAAGACCAAAATTGGTTCAATCTGTTAATGGATTTTTT<br>TCTAAAGGAGCTAATGTTGTTACTCATGGAAGATTTTTTTCTTCTGAACCATCTAAATCTGAAGAT<br>TCTGGATTGGATTTTGTTAAACCAAATAAATGGTTTAATTTGTTGGTTCCAGAATTGTCTTCTTCT<br>GCTAAAATTGCTTTGTCTAGATGGAAAGGAAATGATTTGTTGACTATTCCACCAATGGTTTTGGA<br>AGTTTTTTTGGTTGTTCCAACTGAACATTCTGATTCTTTGGTTTTGAGAGGAGGAGCTGATGGATT<br>GTCTCAAGATGATGTTGTTATTAATGTTGGAAATTATGAAAGAAATGAAATTGTTTTGGAAAGAT<br>GGTTGTTGGAATTTGATTTGACTACTTTGGATAAAAATTCTACTGAAATTTATGGAATTTATAAAA<br>AAATGATTATTTTGTTTAGAAATTTGTATACTTTTGTTAGATTGATGCCAGCTTTTAGATTGTTTCA<br>AGAAGGAAATTGGAAAATTGGAACTAGAACTTTGGATGGATCTGAACCAATTTCTTCTAAAGAT<br>AGAATTGGATTGTCTGATTCTTTTTTGGGAGAATCTAAAAATTCTGAAGAACAAGATCAATCTCA<br>ATGTTATTATTCTCATTTGTCTCAAAAAAAAATTTAGATCTATTACTACTTCTATGGGATCTTTGAAA<br>ATTTCTTGTTCTTTTAGAAGAAATACTGCTTTTAGATGGGCTTCTACTAGAAGAAAAGCTAGAGA<br>TAATACTGTTACTGGAGGATTGTCTAGAGCTGTTGGAATTCAACCATTTAAAACTGGAGTTTTGT<br>CTTCTTCTCCAGGAAGATCTCCAGGACATGGATTTACTGCTACTTTGTGTGCTAGAACTGAATCTC<br>AACCAATTCCATTGCAAATTCATCATAGATCTTCTTCTAATGCTTCTTTGGTTCAATTGTTGAGAAA<br>TCCAAGAAATTCTATTCCAACTTCTATTAATTCTGTTTTGGAAGATCATTCTACTATTTCTCCAGCT<br>ACTAAATTTTCTTCTTCTTTTAGATTGGCTAGAAGAGGATCTTTGCATTCTAGAACTTCTGTTGATA<br>GAAGATCTTCTGATTTGTCTTCTTCTGATATTGATCCAGATCAATTTTATGTTGATGAAGATATTA<br>ATGATTTGATGAGAATGATTGATGCTAGACCAAATTTGAGATTGTCTTCTGCTAGATCTAGAGAA<br>ACTTCTCCATCTTCTTTGAATAGATTTCAATTGTTGCAAAAAACTCATGATATTTTGTCTGATTCTG<br>TTCATGCTTCTTTGCCAACTTCTGCTCATGGAGTTTTGTCTTTGTCTCCATCTAGAAGATATTCTAT<br>TTCTCCACCAACTATGGGACCAGGATCTTCTACTGCTTCTATTTCTCAATCTTTGACTTATGCTAGA<br>ATGGATCATCAAGATTCTGCTCAATCTGCTAATTCTATTAGAGATATTTTGCAATCTTCTTCTAGA<br>AGAAATTCTTCTTCTAATAGAAGAGGATCTGGACAATCTCCAAGACCAGGAACTATTTTGGGATT<br>GCCATCTGGATTGGGATCTGGAGATTCTGCTATTTCTGATGAAGCTTCTAAAGAACATGTTTATG<br>AAGAACATGCTATTATTGATGATGATGAAGAAGAAGAAGATATGATTACTCAAAGAAATTTGAA<br>TCATTTGTTGAAATATAGAAAATTGAGATTGCAAGATACTAAAAAATCTAAAGAAATTACTATTA<br>AAGAAGAAGATGATGATTTGTTGTTTACTATGTCTGATATGAATTTGTCTTGA |
| 0432optC | ATGTCCAAAACAAATTCGACGGTAAAAGGAAGGCCCAAACTAGTTCAATCTGTGAACGGATTTT<br>TTTCCAAAGGTGCGAATGTGGTTACACACGGACGATTTTTTTCATCCGAACCTTCTAAAAGTGAA<br>GATTCAGGTCTGGATTTTGTTAAACCCAATAAATGGTTCAACTTACTGGTTCCAGAACTGTCCTCT<br>AGTGCTAAGATTGCATTATCTCGTTGGAAAGGTAATGACCTACTAACGATCCCACCCATGGTTTT<br>AGAAGTTTTTCTGGTAGTTCCAACAGAGCATTCAGATAGTCTGGTCCTTCGTGGGGGAGCCGAT<br>GGATTGAGCCAAGATGATGTCGTCATCAACGTTGGTAACTACGAGCGCAACGAGATTGTTCTAG<br>AACGATGGTTACTAGAATTTGATTTGACTACCCTGGACAAAAATTCTACTGAGATCTACGGTATT<br>TACAAGAAAATGATCATTCTTTTTCGCAATCTCTACACATTTGTACGATTGATGCCTGCATTTAGG<br>CTCTTTCAAGAGGGGAACTGGAAAATAGGGACTCGAACATTAGATGGTAGCGAGCCAATCTCCA<br>GCAAAGATCGAATTGGTTTAAGTGATTCGTTCCTGGGCGAATCGAAGAATTCAGAAGAGCAAGA<br>TCAAAGTCAGTGCTATTATTCCCATTTGAGCCAAAAAAAATTTCGATCTATAACCACTTCAATGGG<br>TAGTTTGAAGATCAGTTGCAGCTTCCGTAGAAATACTGCCTTTCGGTGGGCAAGCACACGTCGA<br>AAAGCGCGTGATAACACGGTCACAGGTGGGCTTTCTCGGGCTGTTGGAATACAACCTTTCAAGA<br>CTGGTGTTTTATCGTCCAGCCCTGGTCGTAGTCCCGGTCATGGCTTCACTGCGACACTTTGTGCCC |

| | |
|---|---|
| | GGACTGAGTCACAGCCTATCCCTCTTCAAATTCATCATCGCTCAAGCTCTAACGCTTCCCTTGTAC<br>AACTTCTTCGGAATCCAAGAAATAGCATTCCAACGTCAATAAACTCCGTGCTTGAGGACCATTCA<br>ACAATCTCTCCAGCAACAAAATTCTCTTCCTCTTTTAGGTTAGCACGCAGAGGGAGTCTCCATTCC<br>CGAACATCGGTCGATCGTCGTTCAAGTGACTTATCTTCTTCTGATATTGATCCAGATCAATTTTAT<br>GTTGATGAAGATATTAATGATTTGATGAGAATGATTGATGCTAGACCAAATTTGAGATTGTCTTC<br>TGCTAGATCTAGAGAAACTTCTCCATCTTCTTTGAATAGATTTCAATTGTTGCAAAAAACTCATGA<br>TATTTTGTCTGATTCTGTTCATGCTTCTTTGCCAACTTCTGCTCATGGAGTTTTGTCTTTGTCTCCAT<br>CTAGAAGATATTCTATTTCTCCACCAACTATGGGACCAGGATCTTCTACTGCTTCTATTTCTCAATC<br>TTTGACTTATGCTAGAATGGATCATCAAGATTCTGCTCAATCTGCTAATTCTATTAGAGATATTTT<br>GCAATCTTCTTCTAGAAGAAATTCTTCTTCTAATAGAAGAGGATCTGGACAATCTCCAAGACCAG<br>GAACTATTTTGGGATTGCCATCTGGATTGGGATCTGGAGATTCTGCTATTTCTGATGAAGCTTCT<br>AAAGAACATGTTTATGAAGAACATGCTATTATTGATGATGATGAAGAAGAAGAAGATATGATTA<br>CTCAAAGAAATTTGAATCATTTGTTGAAATATAGAAAATTGAGATTGCAAGATACTAAAAAATCT<br>AAAGAAATTACTATTAAAGAAGAAGATGATGATTTGTTGTTTACTATGTCTGATATGAATTTGTCT<br>TGA |

Figure S1

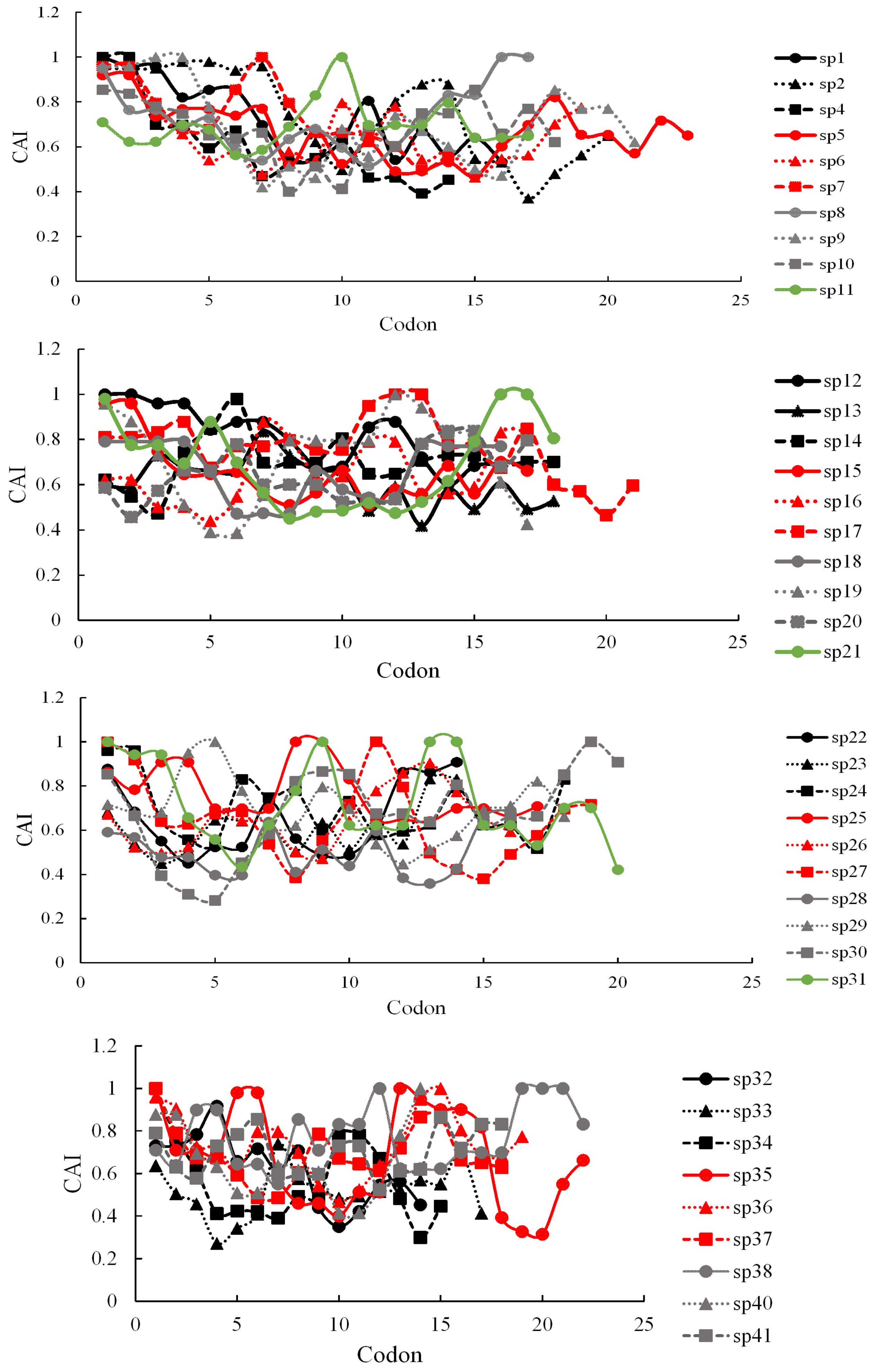

CAI
Codon
sp1
sp2
sp4
sp5
sp6
sp7
sp8
sp9
sp10
sp11
sp12
sp13
sp14
sp15
sp16
sp17
sp18
sp19
sp20
sp21
sp22
sp23
sp24
sp25
sp26
sp27
sp28
sp29
sp30
sp31
sp32
sp33
sp34
sp35
sp36
sp37
sp38
sp40
sp41

Figure S2

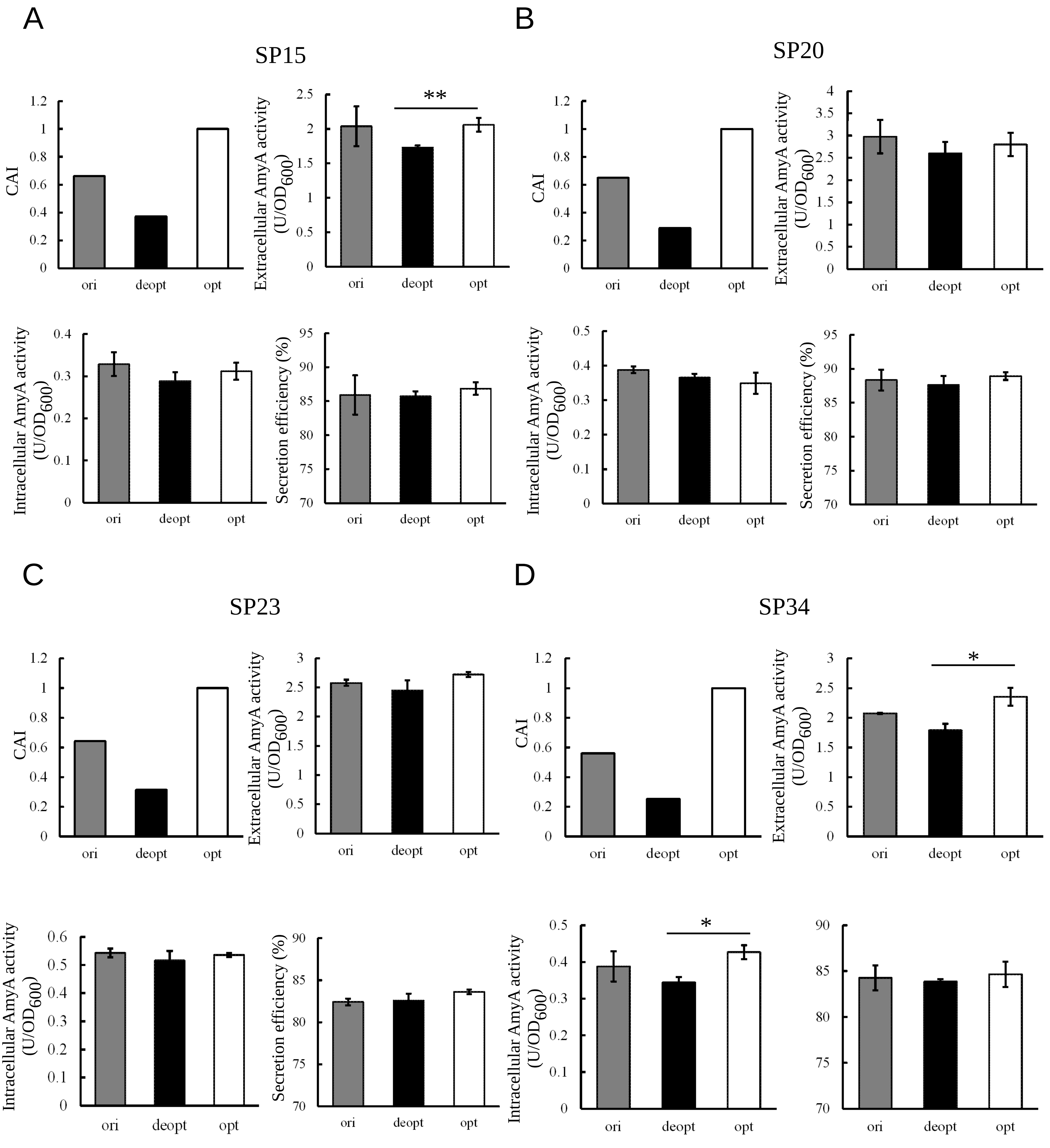

A
SP15
CAI
Extracellular AmyA activity (U/OD$_{600}$)
**
Intracellular AmyA activity (U/OD$_{600}$)
Secretion efficiency (%)
ori
deopt
opt
B
SP20
CAI
Extracellular AmyA activity (U/OD$_{600}$)
Intracellular AmyA activity (U/OD$_{600}$)
Secretion efficiency (%)
ori
deopt
opt
C
SP23
CAI
Extracellular AmyA activity (U/OD$_{600}$)
Intracellular AmyA activity (U/OD$_{600}$)
Secretion efficiency (%)
ori
deopt
opt
D
SP34
CAI
Extracellular AmyA activity (U/OD$_{600}$)
*
Intracellular AmyA activity (U/OD$_{600}$)
*
ori
deopt
opt

Figure S3

A

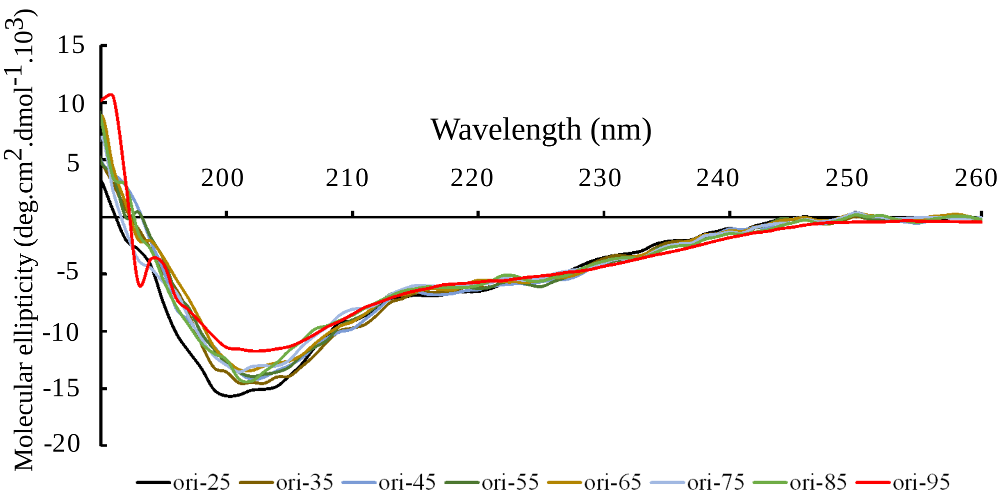
Molecular ellipticity (deg.cm2.dmol-1.103)
15
10
5
-5
-10
-15
-20
Wavelength (nm)
200
210
220
230
240
250
260
ori-25
ori-35
ori-45
ori-55
ori-65
ori-75
ori-85
ori-95

B

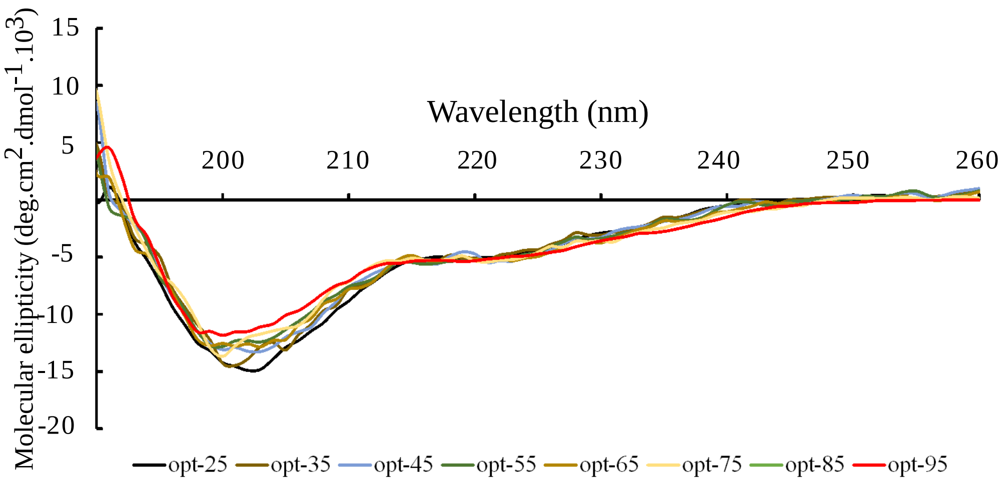
Molecular ellipticity (deg.cm2.dmol-1.103)
15
10
5
-5
-10
-15
-20
Wavelength (nm)
200
210
220
230
240
250
260
opt-25
opt-35
opt-45
opt-55
opt-65
opt-75
opt-85
opt-95
